# RACK1 modulates polyglutamine-induced neurodegeneration by promoting ERK degradation in *Drosophila*

**DOI:** 10.1371/journal.pgen.1009558

**Published:** 2021-05-13

**Authors:** Jun Xie, Yongchao Han, Tao Wang

**Affiliations:** 1 College of Biological Sciences, China Agricultural University, Beijing, China; 2 National Institute of Biological Sciences, Beijing, China; 3 Tsinghua Institute of Multidisciplinary Biomedical Research, Tsinghua University, Beijing, China; Peking University, CHINA

## Abstract

Polyglutamine diseases are neurodegenerative diseases caused by the expansion of polyglutamine (polyQ) tracts within different proteins. Although multiple pathways have been found to modulate aggregation of the expanded polyQ proteins, the mechanisms by which polyQ tracts induced neuronal cell death remain unknown. We conducted a genome-wide genetic screen to identify genes that suppress polyQ-induced neurodegeneration when mutated. Loss of the scaffold protein RACK1 alleviated cell death associated with the expression of polyQ tracts alone, as well as in models of Machado-Joseph disease (MJD) and Huntington’s disease (HD), without affecting proteostasis of polyQ proteins. A genome-wide RNAi screen for modifiers of this *rack1* suppression phenotype revealed that knockdown of the E3 ubiquitin ligase, POE (Purity of essence), further suppressed polyQ-induced cell death, resulting in nearly wild-type looking eyes. Biochemical analyses demonstrated that RACK1 interacts with POE and ERK to promote ERK degradation. These results suggest that RACK1 plays a key role in polyQ pathogenesis by promoting POE-dependent degradation of ERK, and implicate RACK1/POE/ERK as potent drug targets for treatment of polyQ diseases.

## Author summary

Polyglutamine (polyQ) diseases are neurodegenerative diseases caused by the expansion of glutamine repeats in specific proteins. After >100 years of effort trying to decipher the pathogenesis of polyQ diseases, the mechanisms by which these mutant proteins induced neuronal death are still unknown. Here, we performed a genome-wide genetic screen in *Drosophila* for mutations that alleviate polyQ cytotoxicity. We found that a mutation in the gene *rack1*, which encodes a scaffold protein, suppressed polyQ-induced neurodegeneration when polyQ tracts were expresses, and in models of HD and MJD. Importantly, this effect was seen without clearance of the polyQ proteins. Further, we found that RACK1 binds both the E3 ubiquitin ligase POE and ERK, thereby promoting ERK degradation. These data suggest that the RACK1/POE/ERK pathway is involved in pathogenesis of polyQ diseases, and identify RACK1/POE/ERK as potential therapeutic targets for treating polyQ diseases.

## Introduction

A class of neurodegenerative disorders are cause by the expansion of CAG repeats within associated genes, resulting in polyglutamine (polyQ) insertions. There are currently nine types of polyQ diseases, including spinocerebellar ataxias (SCAs) and Huntington’s disease (HD) [1, 2]. One common feature of these polyQ diseases is the formation of polyQ oligomers and aggregates in the cytosol and nucleus [1, 3, 4]. In both human patients and animal models of these diseases, polyQ structures interrupt a variety of cellular functions, including transcriptional regulation, the endoplasmic reticulum (ER), and synaptic dynamics [5–7]. A number of factors were found to reduce the severity of polyQ diseases, primarily by reducing polyQ levels [7–11], but the mechanisms by which polyQ tracts lead to neural degeneration remain unknown.

The Ras/Raf/mitogen-activated protein kinase (MEK)/extracellular signal-regulated kinase (ERK) signaling pathway plays an important role in cellular proliferation, differentiation, and survivival, as well as tumorgenesis [12, 13]. Importantly, ERK signaling is inhibited in two models of polyQ diseases, namely spinocerebellar ataxia type 17 (SCA17) and HD [14–16]. Moreover, ERK activation can protect against cellular dysfunction in cells harboring a mutant form of the gene *huntingtin* (*htt*) [14, 17]. ERK, which is also called Mitogen-activated protein kinase (MAPK), is regulated by the classic Ras/Raf/MEK/ERK signaling cascade [18, 19], but steady-state levels of ERK can also be regulated via alterations in gene expression, protein sub-cellular localization, and protein degradation [19–21]. It has been shown that the deubiquitinase, USP47 (Ubiquitin carboxyl-terminal hydrolase 47) stabilizes ERK by counteracting activities of the E3 ubiquitinases POE (Purity of essence), KCMF1 (Potassium channel modulatory factor 1), and UFD4 (Ubiquitin fusion-degradation 4-like) [22]. However, it is not known whether and how steady-state levels of ERK are controlled in physiological contexts such as polyQ diseases.

Receptor for activated C kinase (RACK1) belongs to the WD repeat family of proteins [23] and functions as a scaffold protein to regulate multiple cellular processes, including translation, immunity, apoptosis, and cancer progression [24–28]. In *Arabidosis thaliana*, RACK1 is involved in regulating MAPK activity [29, 30]. In mammalian cells, RACK1 serves as an adaptor to help activate MAPK JNK (c-Jun N-terminal protein kinase) [31]. RACK1 has been shown to function downstreatm of p38b MAPK in helping to clear aggregates of polyubiquitylated proteins from thoracic muscles of aging flies, thereby promoting proteostasis [32]. RACK1 also reduces cellular toxicity associated with protein aggregates. When overexpressed, it localizes to polyQ aggregates and protects cells from polyQ-induced neurodegeneration [33, 34]. However, in these latter studies they overexpressed human RACK1 in fly models of MJD rather than the endogenous fly RACK1, and the suppression was weak. Moreover, the mechanisms by which RACK1 regulates polyQ toxicity have not been characterized, and it remains unknown whether MAPK signaling is involved in this process.

Here we established a model of polyQ disease in the *Drosophila* eye, and conducted a forward genetic screen to investigate the mechanisms involved in polyQ diseases pathogenesis. Strikingly, loss of RACK1 alleviated polyQ-induced cell death without affecting the formation or clearance of protein aggregates. We then performed a genome-wide RNAi screen to identify genes that could modify the polyQ/*rack1*^*RNAi*^ phenotype. Knockdown of the E3 ligases, POE and KCMF1, further suppressed polyQ-induced cell death, resulting in nearly wildtype looking eyes. By contrast, overexpression of POE abolished the suppressive effects of *rack1* mutation. Further, RACK1 regulated ERK levels post-translationally, as expected, and ERK was indispensable for the role of RACK1 in polyQ-induced neurodegeneration. Finally, we found that RACK1 regulated ERK level by promoting the interaction between ERK and POE. As previous studies found that the ERK pathway is inhibited in polyQ disease models, we hypothesize that the RACK1/POE/ERK pathway plays an important role in polyQ disease pathogenesis, and that this pathway should be considered a target for treating polyQ diseases.

## Results

### Mutating the gene *rack1* alleviated polyQ-induced cellular toxicity

**Fig 1:**
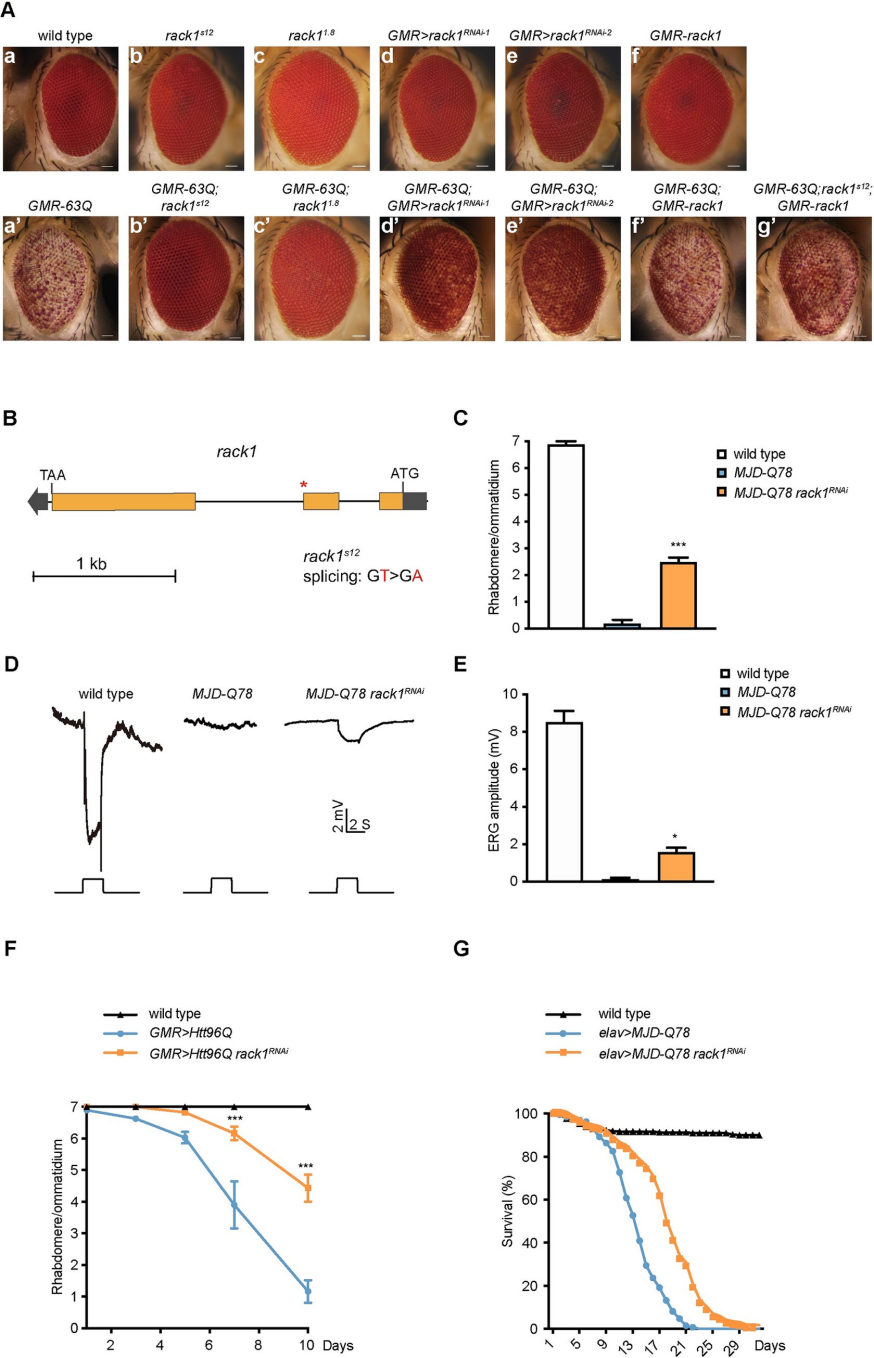
Loss of *rack1* specifically suppresses polyQ toxicity. (A) Light microscope images of eyes of 1-day-old (a) wild type, (a’) *GMR-63Q* (*ey-flp GMR-63Q-HA*), (b) *rack1*^*s12*^ (*ey-flp;GMR-GAL4;rack1*^*s12*^ *FRT40A/GMR-hid CL FRT40A*), (b’) *GMR-63Q;rack1*^*s12*^ (*ey-flp GMR-63Q-HA;rack1*^*s12*^ *FRT40A/GMR-hid CL FRT40A*), (c) *rack1*^*1*.*8*^ (*ey-flp;GMR-GAL4;rack1*^*1*.*8*^ *FRT40A/GMR-hid CL FRT40A*), (c’) *GMR-63Q;rack1*^*1*.*8*^ (*ey-flp GMR-63Q-HA;rack1*^*1*.*8*^ *FRT40A/GMR-hid CL FRT40A*), (d) *GMR*>*rack1*^*RNAi-1*^ (*GMR-GAL4 UAS-rack1*^*KK109073*^), (d’) *GMR-63Q;GMR*>*rack1*^*RNAi-1*^ (*ey-flp GMR-63Q-HA;GMR-GAL4 UAS-rack1*^*KK109073*^), (e) *GMR*>*rack1*^*RNAi-2*^ (*GMR-GAL4;UAS-rack1*^*GD12135*^), (e’) *GMR-63Q;GMR*>*rack1*^*RNAi-2*^ (*ey-flp GMR-63Q-HA;GMR-GAL4;UAS-rack1*^*GD12135*^), (f) *GMR-rack1* (*GMR-rack1/+*), (f’) *GMR-63Q;GMR-rack1* (*ey-flp GMR-63Q-HA;GMR-rack1/+*), and (g’) *GMR-63Q;rack1*^*s12*^*;GMR-rack1* (*ey-flp GMR-63Q-HA;rack1*^*s12*^ *FRT40A/GMR-hid CL FRT40A;GMR-rack1/+*) flies. Scale bar: 50 μm. (B) The *rack1* locus and mutation site (asterisk) associated with *rack1*^*s12*^ allele. (C) Quantification of average number of rhabdomeres per ommatidium in 1-day-old wild type, *MJD-Q78* (*GMR-GAL4/+;UAS-MJD*.*tr-Q78/+*), and *MJD-Q78 rack1*^*RNAi*^ (*GMR-GAL4/+;UAS-MJD*.*tr-Q78/UAS-rack1*^*GD12135*^)flies. At least 20 ommatidia from three flies of each genotype were counted, and significant differences between *MJD-Q78* and *MJD-Q78 rack1*^*RNAi*^ were determined using Student’s unpaired *t* test (n = 3). Data are presented as mean ±SEM. (D-E) ERG recordings (D) and quantification of ERG amplitudes (E) from 1-day-old wild type, *MJD-Q78* (*GMR-GAL4/+;UAS-MJD*.*tr-Q78/+*), and *MJD-Q78 rack1*^*RNAi*^ (*GMR-GAL4/+;UAS-MJD*.*tr-Q78/UAS-rack1*^*GD12135*^) flies. Flies were exposed to a 2-s pulse of orange light after 2 min of dark adaptation, significant differences between *MJD-Q78* and *MJD-Q78 rack1*^*RNAi*^ were determined using Student’s unpaired *t* test (n = 3). Data are presented as mean ±SEM. (F) Quantification of average number of rhabdomeres per ommatidium at indicated days of wild type, *GMR>Htt96Q* (*GMR-GAL4/+;UAS-Htt96Q-eGFP/+*), and *GMR>Htt96Q rack1*^*RNAi*^ (*GMR-GAL4/+;UAS-Htt96Q-eGFP/UAS-rack1*^*GD12135*^) flies. Data are presented as mean ± standard error of the mean (SEM). One-way ANOVA with Tukey’s post hoc test is used to quantify significant differences between results from *GMR>Htt96Q rack1*^*RNAi*^ and *GMR>htt96Q* (n = 3). (G) Lifespan of the wild type, *elav>MJD-Q78* (*elav-GAL4/+;UAS-MJD*.*tr-Q78/+*), and *elav>MJD-Q78 rack1*^*RNAi*^ (*elav-GAL4/+;UAS-MJD*.*tr-Q78/UAS-rack1*^*GD12135*^) flies (n = 200). Data are presented as mean ± standard error of the mean (SEM). P<0.0001, Log-rank test.

Several polyQ disease models have been established in flies by expressing disease-associated proteins with expanded polyQ repeat or polyQ chains alone via the GAL4/UAS system [35–38]. However, to conduct a forward genetic screen to investigate the mechanisms involved in polyQ diseases pathogenesis, we established a simplified model of polyQ disease in the *Drosophila* eye. In this model, we used the *GMR* promoter to express 63 CAG repeats tagged with HA (63Q) in the fly eye. The result was loss of pigment and retinal cells immediately after eclosion (S1A and S1B Fig). When 63Q was expressed in third instar eye discs, it initially localized to the cytosol (in anterior cells), but over time gradually formed aggregates (in posterior cells). By the pupal stage, most 63Q signal was detected in aggregates (S1C Fig). By contrast, 31Q localized to the cytosol in both the third instar eye disc and the pupal eye (S1C Fig). We then subjected this model to EMS mutagenesis to screen for genetic modifiers of 63Q-induced photoreceptor cell death. Because genes involved in polyQ pathogenesis could be essential, we combined the polyQ model with the “*ey-flp/hid*” system to generate flies in which the EMS-induced mutation was homozygous in the eyes [39], but heterozygous in the rest of the animal. Briefly, the *ey-flp/hid* system combines *GMR-hid* and the *FLP/FRT* system. *Ey-flp* induces mitotic recombination of mutated *FRT* containing chromosome arms only in the compound eyes. *GMR-hid* ensures that all eye cells are killed, except for those that are homozygous for your introduced mutation. This allows one to phenotypically screen homozygous loss-of-function mutations in the eye in the F1 generation. Using this strategy, we screened chromosomes 2L, 2R, 3L, and 3R for suppressors of 63Q-induced cell death (Figs 1A, S1D and S8A).

By screening ~100,000 flies for each chromosome arm, we identified ~30 alleles that strongly suppressed polyQ protein-induced cell death. We then excluded mutants that affected transcription mediated by the GMR promoter by checking if these mutants reduced GFP levels of *GMR*-*GFP* flies. The remaining 14 alleles belonged to 4 complementation groups. One complementation group included a single allele, which was homozygous lethal and a strong suppressor of *GMR-63Q* induced cell death.

Using deficiency mapping and genomic DNA sequencing, we located this mutation to the *rack1* genomic locus, which encodes the scaffold protein RACK1 (Receptor for activated C kinase 1). We therefore name this allele *rack1*^*s12*^. This allele contains an RNA splicing mutation at the donor (5’) site (GT to GA) between the second and third exon (Fig 1B), which disrupts RACK1 expression (S2A Fig). A previously reported null allele of *rack1*, *rack1*^*1*.*8*^, also suppressed 63Q-induced cell death (Figs 1A and S8A). Moreover, expressing wild-type *rack1* via the *GMR* promoter restored polyQ-induced cell death in *rack1*^*s12*^ mutants (Figs 1A and S8A). Finally, knocking down *rack1* by RNAi suppressed 63Q-induced cell death, although to a lesser extent, whereas expressing *rack1*^*RNAi*^ alone did not affect eye morphology (Figs 1A and S8A). These results confirmed that *rack1* mutation alleviated polyQ protein-induced cell death.

To determine whether *rack1* modulates specific polyQ disease models, we examined the effect of *rack1* in models of Machado-Joseph disease (MJD) and Huntington’s disease (HD) [40, 41]. Expressing a truncated form of Ataxin-3 with 78 CAG repeats (MJD-Q78) in the fly eye caused a dramatic loss of photoreceptor cells and rhabdomere structures, which are tightly-packed microvilli required for phototransduction [41]. Knocking down *rack1* in this context decreased the amount of photoreceptor cells loss [40] (Figs 1C and S2B and S1 Table). To evaluate photoreceptor function, we performed electroretinography (ERG) to measure electrical responses to light stimulation. At day 1, flies expressing MJD-Q78 exhibited decreased amplitude and loss of on- and off-transients, indicating impaired phototransduction and synaptic transmission [42]. Knocking down *rack1* partially rescued ERG amplitude and restored ERG transients (Fig 1D and 1E). Similarly, knocking down *rack1* alleviated the neurodegeneration associated with mutated Huntington (Htt). Expressing GFP-tagged N-terminal Htt with 96 CAG repeats (Htt96Q) in the eye progressively reduced the number of photoreceptor cells and rhabdomeres. Knocking down *rack1* suppressed this retinal degeneration. In 10 day-old flies, *GMR>Htt96Q* ommatidia generally had only one photoreceptor cell, whereas *GMR>Htt96Q* ommatidia with *rack1*^*RNAi*^ expression had ~4 intact photoreceptor cells (Figs 1F and S2C).

To exclude the possibility that loss of *rack1* suppressed polyQ-induced cell death only in photoreceptor neurons, we used *elav-GAL4* to express MJD-Q78 in all neurons. This greatly shortened lifespan [40]. *elav>*MJD-Q78 flies survived longer when *rack1* was knocked down, indicating that loss of RACK1 suppressed MJD-Q78-induced neurodegeneration (Fig 1G). We next asked whether loss of *rack1* could suppress cell death in models of other neurodegenerative diseases. We modeled retinitis pigmentosa by expressing mutant rhodopsin (*Rh1*^*G69D*^) and tauopathy by expressing *Tau*^*V337M*^ [43, 44]. Importantly, knocking down *rack1* did not affect *Rh1*^*G69D*^- or *Tau*^*V337M*^-mediated retinal cell death (S2D Fig). Taken together, disruption of RACK1 specifically suppressed polyQ-induced neurodegeneration.

### Loss of RACK1 suppressed polyQ toxicity without clearing protein aggregates

**Fig 2:**
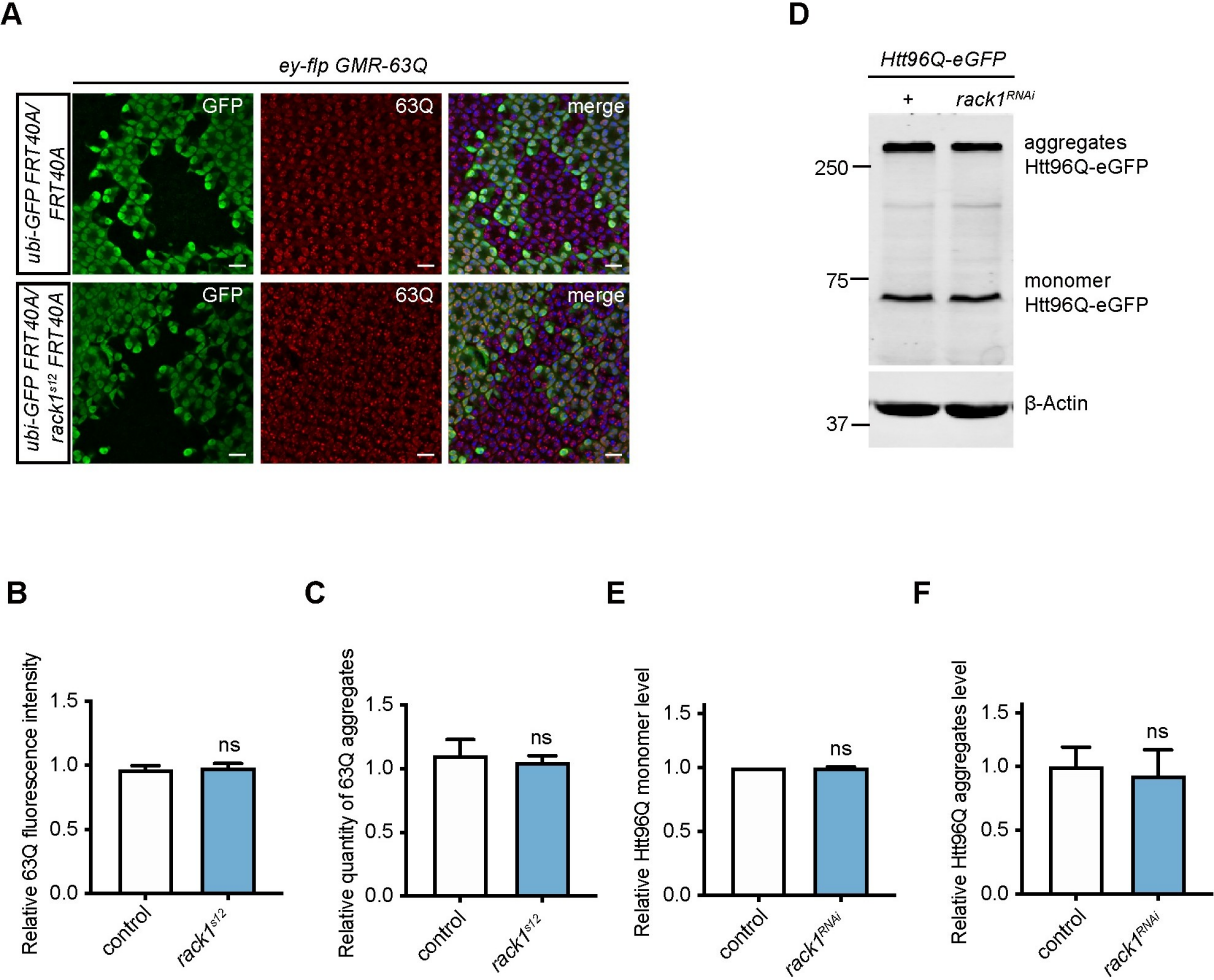
Loss of *rack1* suppresses polyQ toxicity independent of aggregates clearance. (A) Pupa eye staining of control (*hs-flp GMR-63Q-HA;FRT40A/ubi-GFP FRT40A*) and *rack1*^*s12*^ (*hs-flp GMR-63Q-HA;rack1*^*s12*^ *FRT40A/ubi-GFP FRT40A*) mosaic clones by anti-GFP and anti-HA antibodies. GFP negative cells are *rack1* homozygous mutant cells. Scale bar: 10 μm. (B-C) Quantification of relative 63Q fluorescence intensity (B) and quantity of 63Q aggregates (C) in *rack1*^*s12*^ mutant clones compared to GFP-positive control counterparts in panels A (Student’s unpaired *t* test, n = 5). Data are presented as mean ±SEM. (D) Western blot analysis of monomer and aggregate form of Htt96Q proteins extracted from fly eyes with antibody against GFP. β-Actin was used as a loading control. (E-F) Fold-change of Htt96Q monomer (E) and aggregates (F) level in *GMR>Htt96Q*, *rack1*^*RNAi*^ (*GMR-GAL4/+;UAS-Htt96Q-eGFP/UAS-rack1*^*GD12135*^) flies compared to *GMR>Htt96Q* (*GMR-GAL4/+;UAS-Htt96Q-eGFP/+*) flies (n = 3). ns: not significant (Student’s unpaired *t* test). Data are presented as mean ±SEM.

The formation of protein aggregates is a hallmark of polyQ diseases, and most suppressors of polyQ toxicity help to clear these aggregates [9–11]. We next tested whether loss of *rack1* affected the formation of polyQ aggregates. Using the mosaic system in pupal eyes, we found no difference in the levels of 63Q aggregates or total 63Q protein between *rack1*^*s12*^ cells and controls (Fig 2A–2C). We further quantified Htt96Q monomer and aggregate levels by western blot, and found that Htt96Q monomers and aggregates were also not affected by *rack1* knock-down (Fig 2D–2F). Finally, we found that Htt25Q levels were not affected by knocking down *rack1* (S2E and S2F Fig). These results demonstrate that loss of *rack1* did not suppress polyQ toxicity by clearing polyQ aggregates.

RACK1 is a scaffold protein that helps to assemble a variety of signaling molecules involved in multiple processes, including PKC activation, protein translation, and apoptosis [31, 32, 45–47]. To reveal the mechanism by which RACK1 affects 63Q cellular toxicity, we asked whether RACK1 functions through known pathways in this context. RACK1 promotes the release of eukaryotic translation initiation factor 6 (eIF6) from the 60s ribosome by recruiting PKC (protein kinase C) to eIF6 [45]. However, knocking down, knocking out, or overexpressing *pkc53e*, which encodes the major fly PKC, failed to suppress 63Q-induced cell death (S3A Fig), indicating that the classic PKC pathways were not involved in suppressing polyQ toxicity in *rack1* mutants. RACK1 also regulates ribosomal quality control by interacting with ZNF598 [46]. However, knocking down *znf598* did not alleviate 63Q toxicity (S3A Fig). These results indicate that the loss of *rack1* did not suppress polyQ toxicity by regulating translation.

RACK1 interacts with the p38/JNK pathway to regulate apoptosis [31, 32, 47]. However, knocking down or overexpressing JNK, P38a, or P38b did not affect 63Q toxicity or the ability of *rack1*^*RNAi*^ to suppress polyQ toxicity (S3B Fig). Considering that apoptosis is downstream of JNK, we inhibited apoptosis by knocking down apoptosis-related proteins (*rpr*, *hid*, *and grim)* and caspases (*dronc*, *dcp-1*, and *decay*) or by over-expressing Inhibitor of apoptosis (DIAP1). This did not affect 63Q-induced cell death, either with or without *rack1*^*RNAi*^ (S3C Fig). We also assessed the hypoxia pathway and immunity-related proteins, but did not see effects on 63Q toxicity. Therefore, we hypothesized that RACK1 suppresses polyQ-induced neurodegeneration via a novel pathway.

### RACK1 genetically interacted with the E3 ligases POE and KCMF1

**Fig 3:**
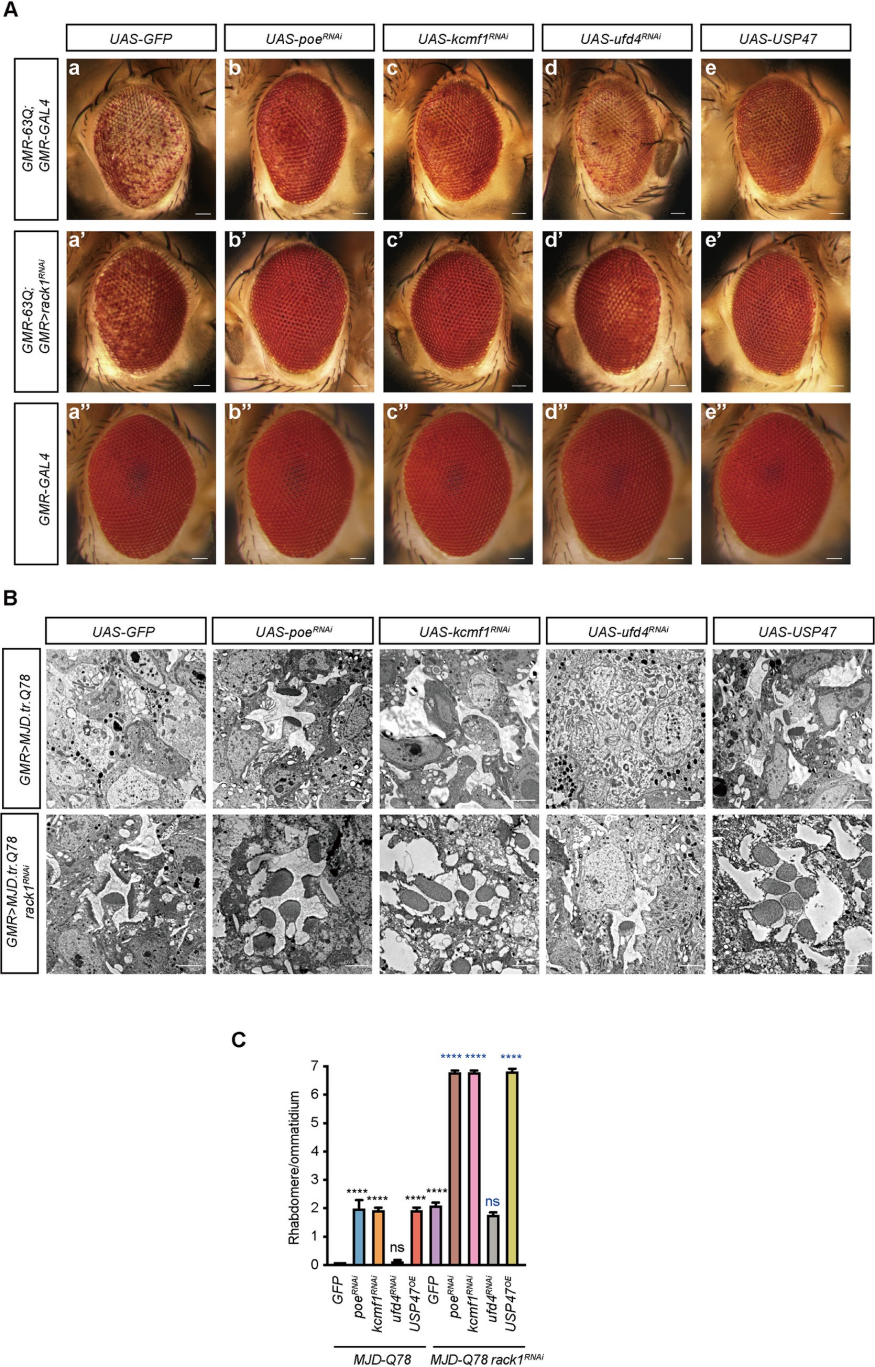
Genome-wide RNAi screening identified POE and KCMF1 as enhancers of *rack1*^*RNAi*^ on suppression of polyQ protein toxicity. (A) Light microscope images of eyes of 1-day-old flies expressing *GFP*, *poe*^*RNAi*^, *kcmf1*^*RNAi*^, *ufd4*^*RNAi*^, and *USP47* in (a-e) *GMR-63Q;GMR-GAL4* (*GMR-63Q-HA;GMR-GAL4*/+), (a’-e’) *GMR-63Q;GMR>rack1*^*RNAi*^ (*GMR-63Q-HA;GMR-GAL4 UAS-rack1*^*KK109073*^/+) and (a”-e”) *GMR-GAL4* (*GMR-GAL4/+*) background. Scale bar: 50 μm. (B) TEM images of eyes of 1-day-old flies expressing *GFP*, *poe*^*RNAi*^, *kcmf1*^*RNAi*^, *ufd4*^*RNAi*^, or *USP47* in either *GMR>MJD-Q78* (*GMR-GAL4/+;UAS-MJD*.*tr-Q78/+*) (upper panels) or *GMR>MJD-Q78 rack1*^*RNAi*^ (*GMR>rack1*^*KK109073*^*/+;UAS-MJD*.*tr-Q78/+*) (lower panels) background. Scale bar: 2 μm. (C) Quantification of average number of rhabdomeres per ommatidium in (B). Data are represented as mean ± SEM (n = 3). P-values of black color represent comparison with *MJD-Q78* flies, p-values of blue color represent comparison with *MJD-Q78 rack1*^*RNAi*^ flies. Significant differences were determined using One-way ANOVA with Tukey’s post hoc test (n = 3).

To determine the pathway through which *rack1* modulated polyQ pathology, we conducted a genome-wide RNAi screen to identify genes that modified the polyQ/*rack1*^*RNAi*^ phenotype. We expressed ~6,000 RNAi lines individually in compound eyes that also expressed *rack1*^*RNAi*^ and *63Q*. We identified more than 300 genes that restored 63Q toxicity when knocked down, but most also induced cell death when expressed alone or combined with 63Q, suggesting effects independent of RACK1. We also identified 14 RNAi lines that further suppressed 63Q-induced cell death in combination with *rack1*^*RNAi*^ (Table 1). Among these 14 were two E3 ligases, POE (pushover or purity of essence) and KCMF1 (zinc-finger protein Potassium Channel Modulatory Factor 1), which each further suppressed polyQ-induced cell death in combination with *rack1*^*RNAi*^, but only slightly suppressed 63Q toxicity when knocked down alone. In contrast, no cell death was detected when *poe*^*RNAi*^ or *kcmf1*^*RNAi*^ were expressed alone (Figs 3A and S8B and S1 Table).

**Table 1:** RNAi alleles that further suppressed polyQ-mediated cytotoxicity in combination with *rack1* knock down.

#TH	Gene name	*63Q;GMR>rack1*^RNAi^ ^*a*^	*63Q;GMR-GAL4* ^*b*^	Function
THU0953	*kcmf1*	Yes	yes	ubiquitin-dependent protein catabolic process; regulating ERK level
THU1137	*poe*	yes	yes	ubiquitin-dependent protein catabolic process; regulating ERK level
THU1058	*vcp*	yes	yes	AAA-ATPase; regulation of programmed cell death
TH01734.N	*herc4*	yes	yes	HECT domain E3 ligase; host defense
THU0683	*hsc70-4*	yes	yes	heat shock protein binding, protein folding
THU1190	*hsc70-2*	yes	yes	heat shock protein binding, protein folding
	*hsp70Ba*			
	*hsp70Bb*			
	*hsp70Bbb*			
	*hsp70Bc*			
THU1287	*hsp70Ba*	yes	yes	heat shock protein binding, protein folding
	*hsp70B*			
	*hsp70Bbb*			
	*hsp70Bc*			
TH03673.N	*CG15739*	yes	yes	dephosphorylation
TH02741.N	*xit*	yes	yes	germ-band extension; protein N-linked glycosylation
TH01799.N	*Gnpnat*	yes	yes	UDP-N-acetylglucosamine biosynthetic process
TH01611.N	*lin*	yes	yes	developmental patterning and cell fate specification
TH04829.N	*βTub56D*	yes	yes	microtubule-based process; muscle attachment
TH01654.N	*vermilion*	yes	yes	protein homotetramerization; tryptophan catabolic process to kynurenine
TH01278.N2	*CG15084*	yes	yes	unknown

a UAS-RNAi lines were crossed with *63Q;GMR>rack1*^*RNAi*^ to identify genes that modified the polyQ/*rack1*^*RNAi*^ phenotype. “yes” indicates genes that when knocked-down enhanced the ability of *rack1*^*RNAi*^ to suppress polyQ-induced cell death.

b UAS-RNAi lines were crossed with *63Q;GMR-GAL4* to identify those that suppressed polyQ-induced cell death. “yes” indicates genes that when knocked-down suppressed polyQ-induced cell death.

Previous studies have shown that POE and KCMF1, together with another E3 ligase UFD4 (Ubiquitin fusion-degradation 4-like), counteract the deubiquitinase USP47 (Ubiquitin Specific Peptidase 47) to destabilize ERK [22]. However, knocking down *ufd4* did not affect 63Q toxicity. This may be because UFD4 had a much weaker effect on ERK levels compared with POE or KCMF1 (Figs 3A and S8B and S1 Table). We then overexpressed USP47 in the compound eyes of *GMR-63Q* flies and found that USP47 suppressed polyQ-induced cell death. Moreover, the suppression of 63Q toxicity mediated by *rack1* knock down was greatly enhanced by overexpressing *USP47* (Figs 3A and S8B). This indicated that *rack1* loss suppressed polyQ toxicity through pathways involving POE/KCMF1/USP47. Given that *rack1* mutants suppressed polyQ-induced cell death independent of aggregate clearance, we next asked whether knocking down *poe*/*kcmf1* or over-expressing *USP47* reduced polyQ aggregates. We generated clones of cells expressing *poe*^*RNAi*^, *kcmf1*^*RNAi*^, or *USP47* in 63Q-expressing eyes with the “flip-out” method [48]. We found that knocking down *poe/kcmf1* or over-expressing *USP47* did not affect 63Q aggregate formation, whereas over-expressing the chaperone, *DnaJ*, significantly reduced polyQ aggregates (S4A and S4B Fig) [36, 49].

**Fig 4:**
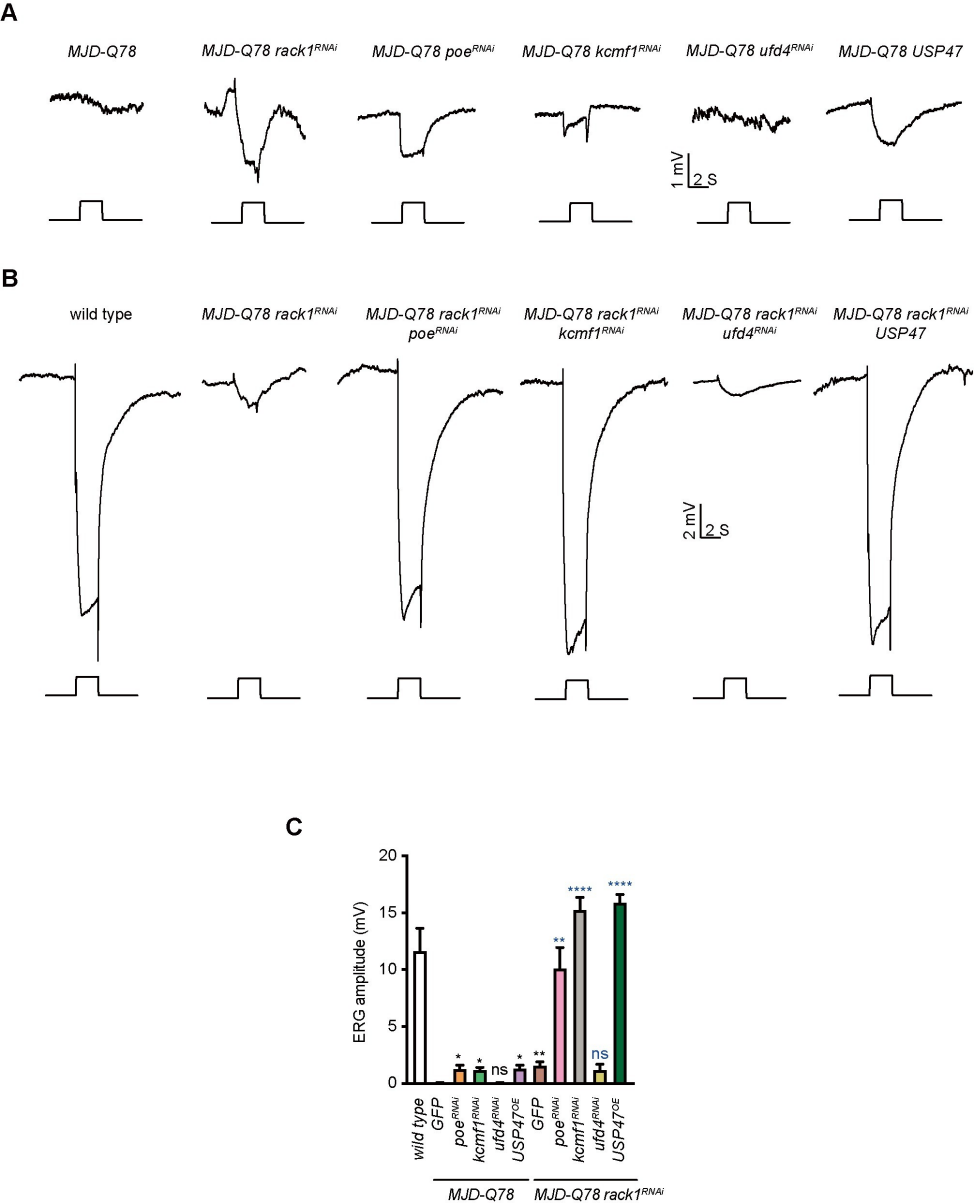
POE and KCMF1 enhanced *rack1*^*RNAi*^ restoration of photoreceptor functions in MJD model. (A) ERG recordings from 1-day-old *MJD-Q78* (*GMR-GAL4/+;UAS-MJD*.*tr-Q78/+*), *MJD-Q78 rack1*^*RNAi*^ (*GMR-GAL4 UAS-rack1*^*KK109073*^*;UAS-MJD*.*tr-Q78/+*), *MJD-Q78 poe*^*RNAi*^ (*GMR-GAL4/+;UAS-MJD*.*tr-Q78/UAS-poe*^*HMS00739*^), *MJD-Q78 kcmf1*^*RNAi*^ (*GMR-GAL4/+;UAS-MJD*.*tr-Q78/UAS-kcmf1*^*HMS00511*^), *MJD-Q78 ufd4*^*RNAi*^ (*GMR-GAL4/+;UAS-MJD*.*tr-Q78/UAS-ufd4*^*RNAi*^), and *MJD-Q78 USP47* (*GMR-GAL4/+;UAS-MJD*.*tr-Q78/UAS-USP47*) flies. (B) ERG recordings from 1-day-old wild type, *MJD-Q78 rack1*^*RNAi*^ (*GMR-GAL4 UAS-rack1*^*KK109073*^*;UAS-MJD*.*tr-Q78/+*), *MJD-Q78 rack1*^*RNAi*^ *poe*^*RNAi*^ (*GMR-GAL4 UAS-rack1*^*KK109073*^*;UAS-MJD*.*tr-Q78/UAS-poe*^*HMS00739*^), *MJD-Q78 rack1*^*RNAi*^ *kcmf1*^*RNAi*^ (*GMR-GAL4 UAS-rack1*^*KK109073*^*;UAS-MJD*.*tr-Q78/UAS-kcmf1*^*HMS00511*^), *MJD-Q78 rack1*^*RNAi*^ *ufd4*^*RNAi*^ (*GMR-GAL4 UAS-rack1*^*KK109073*^*;UAS-MJD*.*tr-Q78/UAS-ufd4*^*RNAi*^), and *MJD-Q78 rack1*^*RNAi*^ *USP47* (*GMR-GAL4 UAS-rack1*^*KK109073*^*;UAS-MJD*.*tr-Q78/UAS-USP47*) flies. Flies were exposed to a 2-s pulse of orange light after 2 min of dark adaptation. (C) Quantification of ERG amplitudes of indicated flies in B and C. Data are presented as mean ± standard error of the mean (SEM). P-values of black color represent comparison with *MJD-Q78* flies, p-values of blue color represent comparison with *MJD-Q78 rack1*^*RNAi*^ flies. Significant differences were determined using One-way ANOVA with Tukey’s post hoc test (n = 3).

We further confirmed the genetic interaction between RACK1 and POE/KCMF1/USP47 in the MJD model. As seen with 63Q, knocking down *poe* or *kcmf1* (or overexpressing *USP47*) alleviated MJD-Q78-induced photoreceptor cells loss. Knocking down *ufd4* had no effect (Fig 3B and 3C). Moreover, we found that either knocking down *poe* or *kcmf1* or overexpressing *USP47* in the *rack1*^*RNAi*^ background largely promoted *MJD-Q78* photoreceptor cell survival compared with the expression of *rack1*^*RNAi*^, *poe*^*RNAi*^, *kcmf1*^*RNAi*^, or *USP47* alone (Fig 3B and 3C). We further evaluated photoreceptor function via ERG recordings. Consistent with the degeneration results, knocking down *poe* or *kcmf1* (or overexpressing *USP47*) increased ERG amplitude for *MJD-Q78* flies, whereas co-expression of *rack1*^*RNAi*^ with *poe*^*RNAi*^, *kcmf1*^*RNAi*^, or *USP47* completely rescued ERG amplitude and transients for *MJD-Q78* flies (Fig 4A–4C). These results suggest that *rack1* alleviated polyQ toxicity through the POE/KCMF1 ubiquitin ligase systems.

### *rack1* mutations suppressed polyQ toxicity through ERK

**Fig 5:**
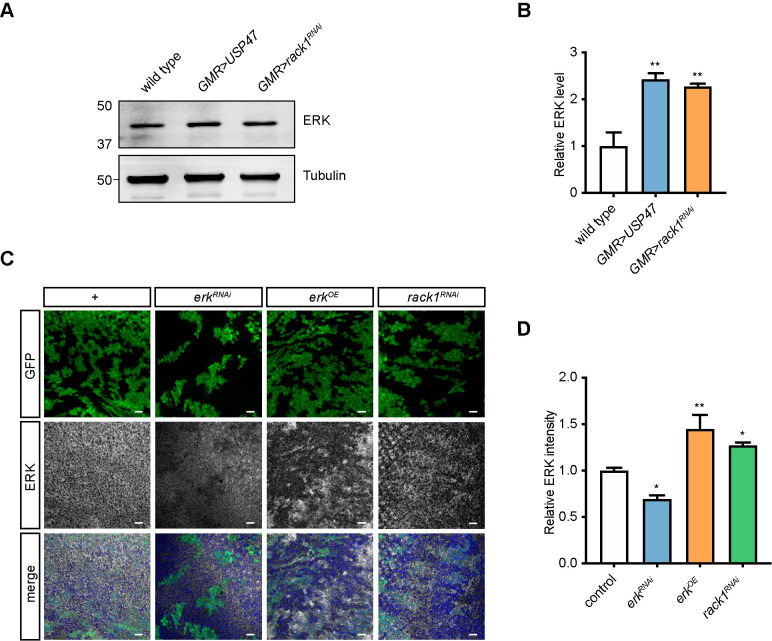
Knocking down *rack1* increased ERK levels. (A) Western blot analysis of proteins extracted from eyes of wild-type (*GMR-GAL4/+*), *GMR>USP47* (*GMR-GAL4/+;UAS-USP47/+*), *GMR>rack1*^*RNAi*^ (*GMR-GAL4 UAS-rack1*^*KK109073*^) flies with antibody against ERK. Tubulin was used as an internal control. (B) Quantification of ERK levels in (A). Data are represented as mean ± SEM, and one-way ANOVA with Tukey’s post hoc test is used (n = 3). (C-D) Heat-shock induced “flip-out” clones expressing *rack1*^*RNAi*^ (*hs-flp;UAS-rack1*^*KK109073*^*/actin>>CD2>>GAL4 UAS-GFP*) increased ERK levels in eye imaginal discs (C). GFP positive cells are *rack1*^*RNAi*^ cells. Flies with *erk*^*RNAi*^ (*hs-flp;;UAS-erk*^*JF01366*^*/actin>>CD2>>GAL4 UAS-GFP*) and *erk* overexpressing (*erk*^*OE*^: *hs-flp;;UAS-erk/actin>>CD2>>GAL4 UAS-GFP*) clones are negative and positive controls, respectively. Scale bar: 10 μm. (D) Quantification of relative ERK intensity between GFP-positive cells and GFP-negative control cells. Data are represented as mean ± SEM, and one-way ANOVA with Tukey’s post hoc test is used (n = 3).

**Fig 6:**
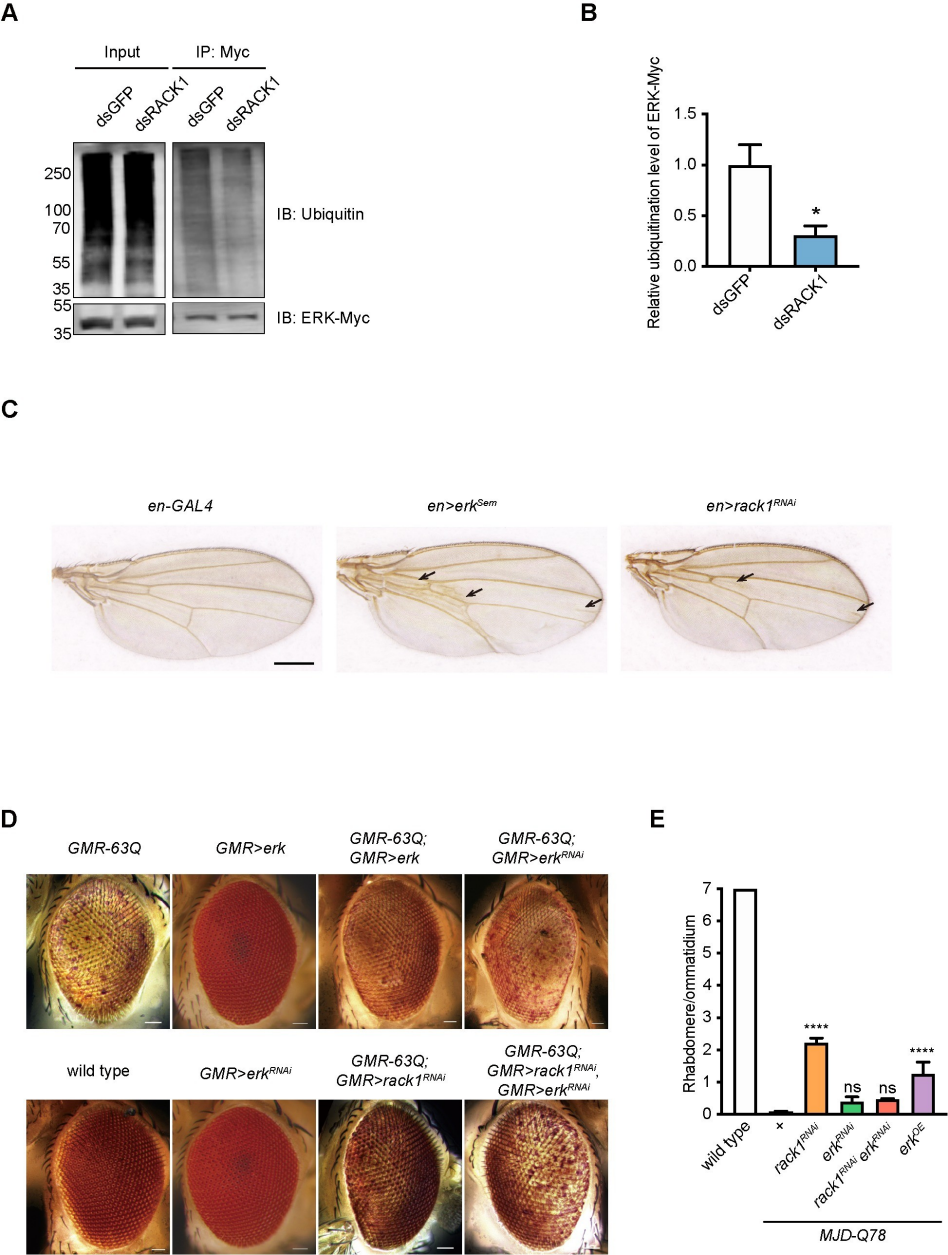
RACK1 regulated ERK levels post-translationally. (A) Shown is western blot analysis of this immunoprecipitation using antibodies against Myc or ubiquitin. (B) Quantification of relative ubiquitination levels of ERK–Myc from (A), data are the mean ± SEM, n = 3. (C) Knocking down *rack1* under the control of *en-GAL4* induced extra wing vein material (indicated by arrows) within the posterior compartment. The expression of *erk*^*Sem*^ served as positive control. Scale bar: 250 μm. (D) Light microscope images of eyes of 1-day-old flies of *GMR-63Q* (*ey-flp GMR-63Q-HA*), *GMR>erk* (*GMR-GAL4/+;UAS-erk/+*), *GMR-63Q;GMR>erk* (*ey-flp GMR-63Q-HA;GMR-GAL4/+;UAS-erk/+*), *GMR-63Q;GMR>erk*^*RNAi*^ (*ey-flp GMR-63Q-HA;GMR-GAL4/+;UAS-erk*^*JF01366*^*/+*), wild-type (*GMR-GAL4/+*), *GMR>erk*^*RNAi*^ (*GMR-GAL4/+;UAS-erk*^*JF01366*^*/+*), *GMR-63Q;GMR*>*rack1*^*RNAi*^ (*ey-flp GMR-63Q-HA;GMR-GAL4 UAS-rack1*^*KK109073*^*/+*), *GMR-63Q;GMR>rack1*^*RNAi*^ *erk*^*RNAi*^ (*ey-flp GMR-63Q-HA;GMR-GAL4 UAS-rack1*^*KK109073*^*/+;UAS-erk*^*JF01366*^*/+*) flies. Scale bar: 50 μm. (E) Quantification of average number of rhabdomeres per ommatidium of wild type, *MJD-Q78* (*GMR-GAL4/+;UAS-MJD*.*tr-Q78/+*), *MJD-Q78 rack1*^*RNAi*^ (*GMR-GAL4 UAS-rack1*^*KK109073*^*;UAS-MJD*.*tr-Q78/+*), *MJD-Q78 erk*^*RNAi*^ (*GMR-GAL4/+;UAS-MJD*.*tr-Q78/UAS-erk*^*JF01366*^), *MJD-Q78 rack1*^*RNAi*^ *erk*^*RNAi*^ (*GMR-GAL4 UAS-rack1*^*KK109073*^*;UAS-MJD*.*tr-Q78/UAS-erk*^*JF01366*^), *MJD-Q78 erk*^*OE*^ (*GMR-GAL4/+;UAS-MJD*.*tr-Q78/UAS-erk*). Transmission electron microscopy images from three one-day-old flies of each genotype were used for quantification. Data are represented as mean ± SEM, and significant differences compared with *MJD-Q78* were determined using one-way ANOVA with Tukey’s post hoc test (n = 3).

Since ERK was the substrate of the POE/KCMF1 E3 ubiquitin ligase, we speculated that loss of *rack1* suppresses polyQ toxicity by regulating levels of ERK. We first measured ERK levels in adult flies and found that overexpressing *USP47* or knocking down *rack1* increased ERK levels by two-fold (Fig 5A and 5B). We then used the “flip-out” system to express *rack1*^*RNAi*^ in GFP-positive cells in eye imaginal discs. Comparing ERK levels between *rack1*^*RNAi*^ cells and neighboring wild-type cells, we further confirmed that down-regulating *rack1* increased ERK levels (Fig 5C and 5D). By contrast, *rack1*^*RNAi*^ and USP47 overexpression did not impact *erk* mRNA levels (S6A Fig). Moreover, we found that knocking down *rack1* also increased ERK levels when 63Q was expressed. Similar results were seen when *poe* or *kcmf1* was knocked down, but not *ufd4* (S5A and S5B Fig). Knocking down *rack1* also did not change the rate of *erk* translation, as measured by polysomal loading of *erk* mRNA transcripts (S6B Fig). We also found that knocking down *rack1* in S2 cells decreased ERK ubiquitination level (Fig 6A and 6B). Finally, knocking down *rack1* using *engrailed-GAL4* (*en-GAL4*) induced extra wing vein material within the posterior compartment, a phenotype consistent with *erk* activation (Fig 6C) [50]. Thus, RACK1 negatively regulated ERK protein levels post-translationally.

Consistent with the up-regulation of ERK by loss of *rack1*, overexpressing *erk* suppressed both 63Q- and MJD-Q87-associated toxicities (Figs 6D, 6E and S8C). To further prove that the suppression of polyQ toxicity by *rack1* loss depended on ERK, we knocked down *erk* using *erk*^*RNAi*^ and found that *erk*^*RNAi*^ prevented *rack1*^*RNAi*^ from suppressing both 63Q- and MJD-Q87-induced cell death (Figs 6D, 6E and S8C). Finally, we found that the knock down or overexpression of *erk* did not affect polyQ levels (S4A and S4B Fig). We also assessed ERK levels in polyQ disease models and found that expressing 63Q or MJD-78Q did not affect ERK levels (S6C and S6D Fig). These results strongly indicate that the ERK pathway is involved in *rack1*-mediated suppression of polyQ toxicity.

### RACK1 functioned as an adaptor protein to promote the degradation of ERK by POE

**Fig 7:**
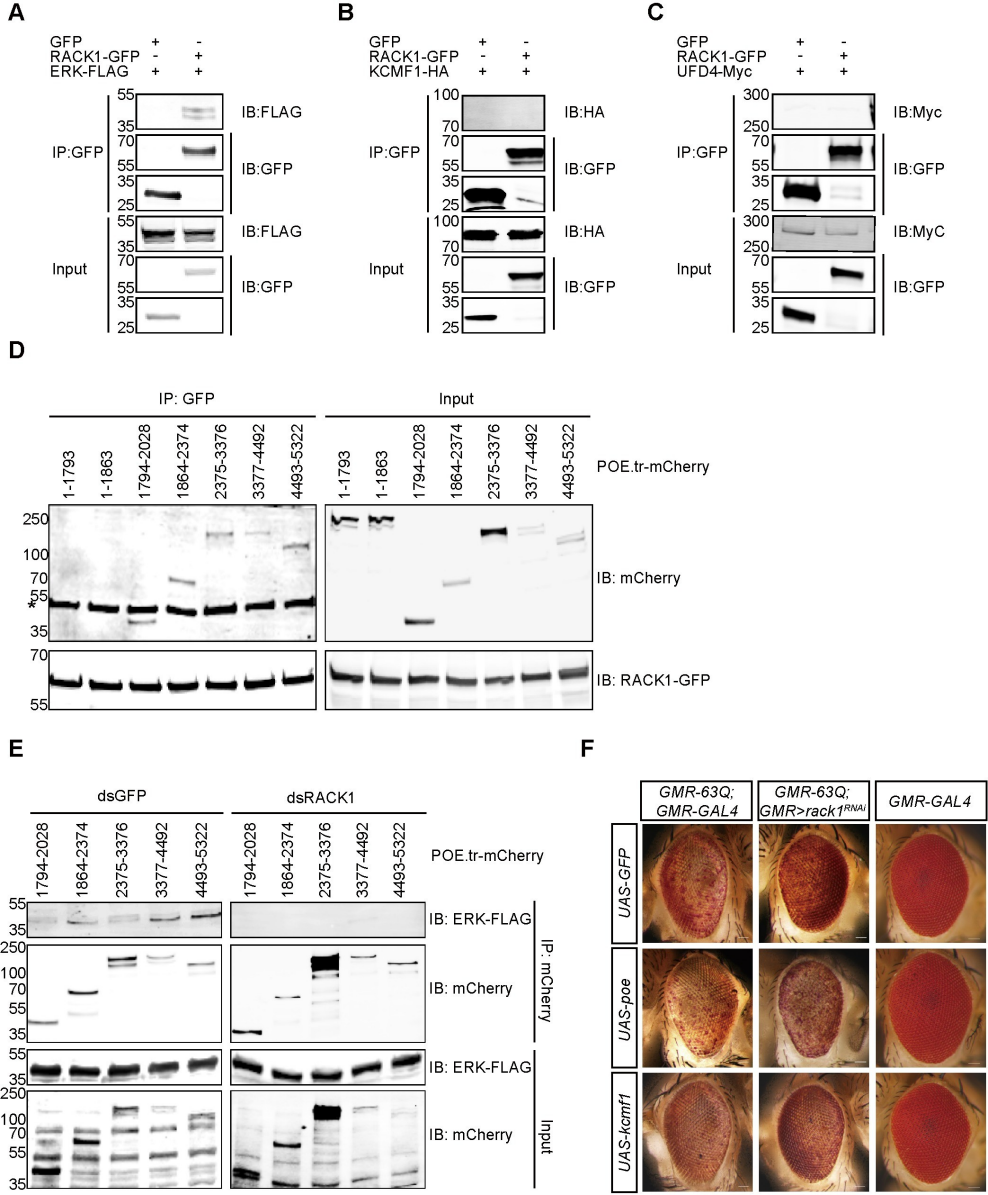
RACK1 scaffolds POE and ERK. (A-C) RACK1 specifically interacts with ERK. FLAG-tagged ERK (A), HA-tagged KCMF1 (B) or Myc-tagged UFD4 (C) was co-expressed in S2 cells with GFP or RACK1-GFP, followed by immunoprecipitation with anti-GFP antibody. (D) Different mCherry-tagged portions of POE were expressed in S2 cells with GFP-tagged RACK1, followed by immunoprecipitation with anti-GFP antibody and western blotting against mCherry. Asterisk indicates unspecific band. (E) S2 cells were treated with dsRNA of *GFP* or *rack1* for two days, followed by co-expression of different mCherry-tagged portions of POE and FLAG-tagged ERK. Cells were lysed and immunoprecipitated with anti-mCherry antibody. (F) Light microscope images show that overexpressing POE reversed suppression of polyQ toxicity by knocking-down *rack1* (*ey-flp GMR-63Q-HA;GMR-GAL4 UAS-rack1*^*KK109073*^*/+;UAS-poe/+*), while overexpressing KCMF1 had no effect (*ey-flp GMR-63Q-HA;GMR-GAL4 UAS-rack1*^*KK109073*^*/+;UAS-kcmf1/+*). Light microscope images of eyes of 1-day-old flies of indicated genotypes. Scale bar: 50 μm.

Because RACK1 is a scaffold protein and regulated ERK levels post-translationally, we hypothesized that RACK1 may recruit E3 ligases to ERK to promote its degradation. To test this hypothesis, we expressed GFP-tagged RACK1 and FLAG-tagged ERK in S2 cells, and found that ERK could be co-immunoprecipitated with RACK1 (Fig 7A). We next assessed the interaction between RACK1 and the E3 ligases, POE/KCMF1/UFD4. Although KCMF1 and UFD4 could not be co-immunoprecipitated with RACK1 (Fig 7B and 7C), POE interacted with RACK1 through multiple binding sites, including the UBR4 domain and the region between the UBR and UBR4 domains (Figs 7D, S7A and S7B). Moreover, as with RACK1, POE co-immunoprecipitated with ERK via multiple binding sites. The interaction between POE and ERK depended on RACK1, since knocking down *rack1* by double-stand RNA (dsRNA) weakened the interaction between POE and ERK (Fig 7E). We also found that overexpressing RACK1 did not enhance the interaction between POE and ERK (S7C Fig). We then overexpressed *poe* using a CRISPR/Cas9-based transcriptional activation system [51] and found that POE abolished the suppression of polyQ-associated cell death by *rack1*^*RNAi*^. By contrast overexpressing *kcmf1* had no effect (Figs 7F and S8D). Finally, overexpression of *poe* reverted ERK levels back to normal in the *rack1*^*RNAi*^ background, while overexpressing *kcmf1* had no effect (S7D and S7E Fig). We conclude that RACK1 functions to stabilize the interaction between ERK and the E3 ligase POE, and that mutations in *rack1* disrupt POE-dependent ERK degradation. These increased levels of ERK then suppress polyQ-associated neurodegeneration.

## Discussion

The fact that polyQ repeats aggregate and are toxic by themselves suggest that they are the critical driver of neurodegenerative disease pathogenesis, rather than the proteins they are associated with in different diseases. High-throughput screens have been performed in multiple systems to identify factors that modify polyQ aggregation [52–54], with most of these modifiers affecting the accumulation or the physical properties of polyQ aggregates [55]. In this study, we generated a simple model of polyQ disease by expressing 63 CAG repeats in the fly eye and conducted a genome-wide, loss-of-function screen to identify suppressors of polyQ-induced cell death. We found that a loss-of-function mutation in *rack1* alleviated the cytotoxicity of polyQ proteins. Importantly, loss of *rack1* did not affect the proteostatic control of polyQ proteins, indicating that RACK1 instead affected cytotoxicity downstream of aggregate formation. Chaperones, including HSP40/DNAJ, protect cells in the context of different polyQ disease models, again indicating that the toxicity of polyQ proteins is derived specifically from the polyQ tracts, not from the unique features of the pathogenic proteins [10, 36, 38, 56]. However, since chaperons reduce levels of both the polyQ tracts and the associated protein, this result is not definitive proof that the polyQ tracts are the primary culprit in mediated neuronal cell death. It has also been proposed that unique features of polyQ proteins also modulate pathogenesis of special polyQ diseases [57, 58]. Our results demonstrate that mutating *rack1* prevented neurodegeneration in a general model of polyQ disease, as well as two models of specific polyQ diseases, namely MJD and HD. This indicates that different polyQ diseases share common pathogenic features.

RACK1 is a seven WD repeat domain protein, and provides a platform for protein-protein interactions [23]. RACK1 helps to regulate stress responses and apoptosis through scaffolding signaling proteins such as PKC, ZNF598, and JNK [31, 45, 46]. However, all these signaling pathways are not required for RACK1 function in the context of polyQ pathogenesis. Here, we demonstrate that RACK1 physically interacted with ERK and negatively regulated ERK levels post-translationally. We provide further genetic evidence that the suppression of polyQ toxicity by *rack1* mutation depended on ERK stabilization. Knocking down *erk* abolished the suppressing effects of *rack1* on polyQ-induced neurodegeneration. Therefore, we present both biochemical and genetic evidence that loss of RACK1 suppressed polyQ-induced cytotoxicity by stabilizing ERK.

POE is an N-terminal UBR box E3 ubiquitin ligase, and regulates ERK stability together with another E3 ligase, KCMF1 [22]. In a genome-wide RNAi screen for modifiers of the *rack1* phenotype, we found that knockdown of POE or KCMF1 further suppressed polyQ toxicity when expressed with *rack1*^*RNAi*^. In contrast, overexpressing their counterpart, the deubiquitinase USP47, suppressed polyQ-induced cell death, and this suppression was improved in combination with *rack1* mutation. It has been suggested that ERK is a substrate for POE, and indeed we found that ERK interacted with POE. Further, both POE and ERK bound RACK1, and interaction between POE and ERK was disrupted by knocking down *rack1*, suggesting that the interaction between POE and ERK depends on their ability to bind RACK1. Furthermore, *rack1* mutation did not suppress polyQ-induced cell death when POE was overexpressed. In contrast, KCMF1 did not interact with RACK1, and, consistent with this, overexpressing *kcmf1* did not reverse the suppression effects of *rack1* mutants on polyQ cytotoxicity. This suggests that KCMF1 may not be the key RACK1 cofactor. Instead, our data indicate that RACK1 plays an essential role in promoting the degradation of ERK by bringing together ERK and the E3 ligase, POE. These results further validate our finding that overexpressing *rack1* does not enhance polyQ toxicity, since excessive scaffolding proteins may not enhance the interaction between POE and ERK.

The ERK pathway is involved in promoting cell survival and the clearance of protein aggregates in some neurodegenerative diseases. While ERK signaling is upregulated in HD cell lines and a mouse model of SCA-17 [14, 17], studies in a fly model of HD and patient fibroblasts indicate that polyQ proteins inhibit ERK signaling [16, 59]. Despite the fact that ERK regulation by polyQ is controversial, a number of studies agree that activating the ERK signaling pathway protects against polyQ toxicity [14, 16, 17, 59]. Further studies utilizing the SCA1 model suggest that ERK may help prevent polyQ aggregation [60]. Consistent with the role of ERK in cell survival, we found that ERK signaling protected against polyQ toxicity, both in models of general polyQ disease and models of specific polyQ diseases, namely MJD and HD. This suggests that ERK protects against polyQ disease pathogenesis. Moreover, upregulating levels of ERK protein through the genetic manipulation of POE, USP47, or RACK1 prevented polyQ-induced cell death in MJD and HD models. Although the mechanisms by which ERK signaling regulates polyQ toxicity remain unknown, our results suggest that ERK suppresses polyQ-induced cell death downstream of aggregate formation. Moreover, ERK levels was not reduced in polyQ-expressing cells, indicating that inhibition of ERK pathway polyQ is not directly involved in the pathogenesis of polyQ toxicity.

Because the abnormal expansion of polyQ repeats plays a pivotal role in pathogenesis of polyQ diseases, clearing polyQ aggregates via molecular chaperones has been extensively studied as a strategy for treating polyQ diseases. However, these types of treatment regimens could impair general cellular proteostasis, as many polyQ containing proteins play essential roles. Here we show that the RACK1/POE/ERK signaling pathway does not clear polyQ protein aggregates, but instead specifically ameliorates late pathogenic events associated with polyQ diseases. Our data thus indicate that the RACK1/POE/ERK pathway is a potential therapeutic target for treating polyQ diseases.

## Methods and materials

### Fly stocks

The following fly strains were obtained from the Bloomington Stock Center: (1) *GMR-GAL4* [61], (2) *elav-GAL4* [62], (3) *UAS-MJD*.*tr-Q78* [40], (4) *UAS-Htt96Q-eGFP* [41], (5) *UAS-GFP*, (6) *UAS-mCD8-GFP* [63], (7) *UAS-p38b* [64], (8) *UAS-JNK* [65], (9) *GMR-DIAP1* [66], (10) *GMR-Tau*^*V337M*^ [44], (11) *Df(3L)H99*, (12) *P[TRiP*.*HMS01779]attP2* (*dcp-1*^*RNAi*^), (13) *P[TRiP*.*HMC06381]attP40* (*znf598*^*RNAi*^), (14) *P[TRiP*. *HMS01224]attP2* (*p38a*^*RNAi*^), (15) *M(vas-int*.*Dm)ZH-2A;M(3xP3-RFP*.*attP)ZH-86Fb*, (16) *M(vas-int*.*Dm)ZH-2A;M{3xP3-RFP*.*attP}ZH-51C*, (17) *w*^*1118*^, (18) *rack1*^*1*.*8*^ *FRT40A/CyO*. The following fly RNAi strains were obtain from the Tsinghua Fly Center: (1) *P[TRiP*. *HMS00739]attP2* (*poe*^*RNAi*^), (2) *P[TRiP*. *HMS00511]attP2* (*kcmf1*^*RNAi*^), (3) *P[TRiP*. *JF01366]attP2* (*erk*^*RNAi*^), (4) *P[TRiP*. *SH05382*.*N]attP40* (*rpr*^*RNAi*^), (5) *P[TRiP*. *SH05271*.*N]attP2* (*decay*^*RNAi*^), (6) *P[TRiP*. *JF02641]attP2* (*pkc53e*^*RNAi*^), (7) *P[TRiP*. *GL00140]attP2* (*p38b*^*RNAi*^), (8) *P[TRiP*. *SH05212*.*N]attP2* (*jnk*^*RNAi*^), (9) *P[TRiP*. *HMS00758]attP2* (*dronc*^*RNAi*^). The *P[KK109073]VIE-260B* (*rack1*^*RNAi*^) and *P[GD12135]v27858* (*rack1*^*RNAi*^) flies were obtained from Vienna Drosophila Resource Center [67]. The knock-down efficiency of RNAi lines were verified by qPCR (S1 Table). *UAS-p38a* and *UAS-kcmf1* were obtained from the flyORF collection of the Zurich ORFeome Project [68]. The following flies were maintained in the laboratory of T. Wang: (1) *UAS-dnaJ-1*, (2) *hs-flp;UAS-Dcr-2;actin>>CD2>>GAL4 UAS-GFP*, (3) *GMR>Rh1*^*G69D*^, (4) *hs-flp;ubi-GFP FRT40A*, and (5) *ey-flp ninaE-Rh1-GFP;GMR-hid CL FRT40A /CyO*. For animal studies, no randomization and no blinding were used.

### Generation of plasmid constructs

The *rack1*, *erk*, and *kcmf1* cDNAs were amplified from the cDNA clones RE74715, RE08694, and LP17815, respectively, which were obtained from the *Drosophila* Genomic Resource Center (DGRC). The *ufd4* and truncated *poe* cDNAs were reverse transcribed and amplified from total RNA extracted from *w*^*1118*^ flies. To construct *pIB-rack1-GFP*, the entire coding sequence of *rack1* was subcloned into the *pIB-cGFP* vector between the *EcoRI* and *NotI* sites. To construct *pIB-erk-FLAG*, the *3XFLAG* tag was subcloned into the *pIB* vector between the *BamHI* and *XbaI* sites, followed by subcloning *erk* cDNA between the *EcoRI* and *NotI* sites. To construct *pIB-poe*.*tr-mCherry*, the *mCherry* tag was subcloned into the *pIB* vector between the *BamHI* and *XbaI* sites, and the specific truncated coding sequences of *poe* were subcloned into the *pIB-mCherry* vector between the *EcoRI* and *NotI* sites. To construct *pIB-kcmf1-HA*, the *3XHA* tag was subcloned into the *pIB* vector between the *BamHI* and *XbaI* sites, followed by subcloning the entire coding sequence of *kcmf1* between the *EcoRI* and *NotI* sites. To construct *pIB-ufd4-Myc*, the *Myc* tag was subcloned into the *pIB* vector between the *BamHI* and *XbaI* sites, and the entire coding sequence of *ufd4* were subcloned into *pIB-Myc* vector between the *EcoRI* and *NotI* sites.

### Generation of transgenic flies

To generate the *pGMR-63Q-HA* construct, a HA tag was added to the C-terminus of the 63Q cDNA and subsequently subcloned into the *pGMR* vector [69]. The construct was injected into *w*^*1118*^ embryos, and transgenic insertions on the X-chromosome were maintained. To generate *GMR-63Q-Myc* flies, the 63Q cDNA with a C-terminal Myc tagged was subcloned into the *pGMR-attB* vector, and the construct was injected into *M(vas-int*.*Dm)ZH-2A;M{3xP3-RFP*.*attP}ZH-51C* embryos. To generate *GMR-rack1* flies, *rack1* cDNA was subcloned into the *pGMR-attB* vector, and the construct was injected into *M(vas-int*.*Dm)ZH-2A;M{3xP3-RFP*.*attP}ZH-86Fb* embryos. To generate *pUAST-attB-pkc53e* construct, *pkc53e* cDNA was amplified from the cDNA clone GH03188 from DGRC, and subcloned into the *pUAST-attB* vector. The construct was injected into *M(vas-int*.*Dm)ZH-2A;M{3xP3-RFP*.*attP}ZH-86Fb* embryos to generate *UAS-pkc53E* flies. To generate *UAS-USP47* flies, *USP47* cDNA was amplified from the cDNA clone LD26783 from DGRC and subcloned into the *pUAST-attB* vector, followed by injected into *M(vas-int*.*Dm)ZH-2A;M{3xP3-RFP*.*attP}ZH-86Fb* embryos. To generate *UAS-erk* flies, *erk-FLAG* sequence from the *pIB-erk-FLAG* clone was subcloned into the *pUAST-attB* vector, and the construct was injected into *M(vas-int*.*Dm)ZH-2A;M{3xP3-RFP*.*attP}ZH-86Fb* embryos. All transformants were identified on the basis of eye color.

The *ufd4*^*RNAi*^ line was generated as described [70]. Briefly, a designed two 23nt short hairpin RNA sequences (GCTGTGCTGCTAGATATTTGT) targeting the coding region of *ufd4* was cloned into a VALIUM20 vector. The plasmids were then injected into *M{vas-int*.*Dm}ZH-2A;M{3xP3-RFP*.*attP}ZH-86Fb* embryos, and transformants were identified on the basis of eye color.

*poe* was overexpressed using the flySAM system [51]. Briefly, one sgRNA (TGGCTCCAGCTTGACGTCGG) targeting 350 base pairs upstream of the transcription starting site of *poe* was cloned into the flySAM vector. Plasmids were then injected into *M{vas-int*.*Dm}ZH-2A;M{3xP3-RFP*.*attP}ZH-86Fb* embryos, and transformants were identified on the basis of eye color, and further crossed with *GMR-GAL4* flies to overexpress *poe*.

### Generation of *pkc53e* mutant flies

The *pkc53e*^*ko*^ mutations were generated using the Cas9/sgRNA system [68]. Briefly, a pair of guide RNAs targeting the *PKC53E* locus were designed (sgRNA1: GCGGATGCGATCACACGGAG, sgRNA2: ACCATCGCAAAAGAAGCCGA) and cloned into the *U6b-sgRNA-short* vector. Plasmids were injected into the embryos of *nos-Cas9* flies, and deletions were identified by PCR using the following primers: forward primer 5’-CGAGTGCTATGTTCCACTTCAACA-3’ and reverse primer 5’-TCGTCTGAAAGCAGGAGTGTAATT-3’.

### Objective criteria for scoring retinal phenotypes

All genotypes presented here exhibited highly uniform retinal phenotypes. We examined eye phenotypes of ≥100 flies per genotype, and phenotypes shown in the images (obtained via light microscopy) are present in 100% of the animals. For genetically identical flies, there was no appreciable phenotypic variability. In cases where enhancement or suppression is reported, this phenotype was present in 100% of the animals. Nevertheless, to apply quantitative analyses, we scored the severity of eye phenotypes, as previously reported [71].

### EMS screening

The EMS screening were performed as described [72]. Briefly, the second chromosome of *FRT40A* or *FRT42D* flies and the third chromosome of *FRT2A* or *FTR82B* flies were isogenized, and young male flies were fed 25 mM EMS (Sigma, St. Louis, MO) in 2% sucrose for 8 h. Flies were then mated immediately to *ey-flp*,*GMR-63Q-HA;GMR-hid CL FRT40A/CyO*, *ey-flp*,*GMR-63Q-HA;GMR-hid CL FRT42D/CyO*, *ey-flp*,*GMR-63Q-HA;GMR-hid CL FRT2A/TM3* and *ey-flp*,*GMR-63Q-HA;GMR-hid CL FRT82B/TM3* flies. F1 progenies were screened by suppression of pigment loss, and approximately 100,000 F1 flies were screened for each chromosome arm (S1D Fig).

### Electroretinogram recordings

The electroretinogram experiments were performed as described [69]. Briefly, two glass microelectrodes were filled with Ringer’s solution; one was placed on the surface of the compound eye, and one was placed on the thorax. The light source intensity was ~2000 lux, and the light color was orange (source light was filtered using a FSR-OG550 filter). ERG signals were amplified with a Warner electrometer IE-210, and recorded with a MacLab/4 s A/D converter and the clampelx 10.2 program (Warner Instruments, Hamden, USA).

### Transmission electron microscopy (TEM) and retinal degeneration analysis

Transmission electron microscopy was performed as described [73]. Briefly, adult fly heads were dissected, fixed, dehydrated, and embedded in LR White resin. Thin sections (80 nm) were prepared at a depth of 30–40 μm, then stained with uranyl acetate and lead citrate (Sigma, St. Louis, MO) and examined using a JEOL JEM-1400 transmission electron microscope (JEOL Ltd., Tokyo, JAPAN). For retinal degeneration analysis, flies were raised at 25°C in a 12 h light/12 h dark cycle, and collected at indicated ages. More than 20 ommatidia were counted for each eye section, and more than three flies per genotype were scored.

### Lifespan assay

Lifespan assay were performed as described [74]. Briefly, ~200 newly enclosed female flies of each genotype were collected and placed in groups of 20 individuals. Flies were kept at 25°C with 65% humidity under 12 h light/12 h dark cycles. Flies were transferred to new vials every 2 days, and the number of dead flies was counted every day.

### Immunofluorescence analysis

Immunofluorescence labeling was performed as described [39]. Eye imaginal discs or pupa eyes were dissected in PBS solution (pH 6.8) and fixed in 4% paraformaldehyde in PBS buffer for 30 min. Eye discs or pupa eyes were incubated with diluted antibodies targeting GFP (rabbit, 1:200, Invitrogen), HA (rat, 1:200, Roche), ERK (rabbit, 1:200, Cell Signaling Technology), and Myc (rabbit, 1:200, Sigma). Anti-rabbit and rat IgG antibodies labeled with Alexa Fluor 488, 568, or 647 (1:500, Invitrogen) were used as secondary antibodies. Images were captured with a Nikon A1-R confocal microscope (Nikon, Tokyo, Japan). Acquired images were processed using Photoshop CC 2017 and NIS-Elements AR Analysis 5.20.00.

### Western blot analysis

Western blotting was performed as described [39]. Briefly, 20 fly eyes were dissected and homogenized in SDS sample buffer with a Pellet Pestle (Kimble/Kontes). The proteins were fractionated by SDS-PAGE and transferred to Immobilon-P transfer membranes (Millipore) in Tris-glycine buffer. The blots were probed with Mouse Tubulin primary antibodies (1:2000 dilution, Developmental Studies Hybridoma Bank), Rabbit ERK (1:1000, Cell Signaling Technology), Mouse β-Actin primary antibodies (1:1000 dilution, Santa Cruz Biotechnology) and Rabbit RACK1 antibodies (1:1000 dilution, Dr. J. Kadrmas lab) followed by IRDye 680 goat anti-Rabbit IgG (LI-COR) and IRDye 800 goat anti-Mouse IgG (LI-COR) as the secondary antibodies. The signals were detected with the Odyssey infrared imaging system (LI-COR).

### Analysis of polyQ aggregates

For mosaic experiments, *GMR-63Q-HA;rack1*^*s12*^ *FRT40A/CyO* flies or *GMR-63Q-HA;FRT40A* flies were crossed with *hs-flp;ubi-GFP FRT40A* flies, and heat shocked at the 1^st^ instar larva stage at 37°C for 1 hour to induce mosaic clone. Pupa eyes were dissected at 36~40 h APF (after pupa formation), and labeled using antibodies against HA (1:200, Roche). For calculation, GFP-positive cells are control cells, and GFP negative cells are mutant cells. For flip-out experiments, flies overexpressing (UAS lines) or knocking down (RNAi lines) specific genes were crossed with *hs-flp;GMR-63Q-Myc;act>>CD2>>GAL4 UAS-GFP/SM-TM* flies, and pupa eyes were dissected at 36~40 h APF, followed by labeling using anti-Myc antibodies (1:200, Sigma). For one pupa eye slide, GFP-positive cells are RNAi or overexpressing cells, and GFP negative cells are control cells. For each slide, five non-overlapping areas from GFP negative cells and GFP positive cells were randomly picked, and both the intensity and quantity of aggregates were counted by NIS-Elements AR Analysis 5.20.00 software.

### Cell culture, dsRNA synthesis, and immunoprecipitation

S2 cells were grown at 26°C in Schneider’s Drosophila medium (Sigma- Aldrich) with 10% fetal bovine serum (Gibco BRL), dsRNA was synthesized *in vitro* and transfected as reported [75]. Plasmids were transfected using Vigofect reagent (Vigorous Biotechnology), and cells were collected and lysed with 10 mM Tris-HCl lysis buffer (pH 7.4, 150 mM NaCl, 0.5 mM EDTA, 0.5% NP-40, 25 mM NaF, and 1 mM Na_3_VO_4_ with 1× proteinase inhibitor cocktail [Sigma-Aldrich]). S2 cells were pre-treated with dsRNA against *GFP* or *rack1* for four days, and then transfected with plasmids for two days. To inhibit proteasome activity, cells were treated with 5 μM MG132 for two days. Immunoprecipitations were performed with mCherry beads (Chromotek) and GFP beads (Chromotek). The bound proteins were analyzed by western blotting against Rabbit GFP antibodies (1:1000 dilution, Torrey Pines Biolabs), Mouse FLAG antibodies (1:2000 dilution, Sigma), Rat HA antibodies (1:1000 dilution, Roche), Rabbit Myc antibodies (1:1000 dilution, Sigma), and Rabbit mCherry antibodies (1: 1000 dilution, Biovision).

### Quantitative RT-PCR

Quantitative RT-PCR was performed as described [76]. Total RNA was prepared with Trizol reagent (Invitrogen) from dissected fly eyes, and was treated with TURBO DNase (ThermoFisher). The cDNA was synthesized with RT master mix (RR036A-1; Takara). An iQ SYBR green supermix (Bio-Rad) was used for real-time PCR, and results were analyzed with a CFX96 Real-Time PCR Detection System (Bio-Rad).

The following primers were used for this study: ERK: forward, 5′-TGCACATCCCTATTTAGAGCAA-3′; reverse, 5′-AATGGCACTTCAGCGACAGCC-3′; Rpl32: forward, 5′-GCCGCTTCAAGGGACAGTATCTG-3′; reverse, 5′-AAACGCGGTTCTGCATGAG-3′; RACK1: forward, 5′-TCAGGTGGCAAGGACTCCAAG-3′; reverse, 5′-GCACAGGGCGTTGATGATGTCGTT-3′; POE: forward, 5′-TCAGCAAGCTACATGACCTTAGA-3′; reverse, 5′-TGCACAAGTCGAATGTTCCT-3′; KCMF1: forward, 5′-AATACAAACAGAACGCTAGTGCAG-3′; reverse, 5′-TGTCGGGAATTTGATTTGTG-3′; UFD4: forward, 5′-AATTACCAGGGACCGTAGAGG-3′; reverse, 5′-TTCGCGTCCTCCATAGGAT-3′.

### Polysome fractionation

Polysome fractionation was performed as described [77]. S2 cells were cultured in dsRNA for four days and then treated with cycloheximide (CHX) at 0.1 mg/ml for 30 min. For each RNAi, 10^8^ cells were pelleted and washed once with cold PBS containing 0.1 mg/ml CHX. The cells were then lysed in polysome lysis buffer (PLB; 20 mM Tris-Cl, pH 7.5, 250 mM KCl, 10 mM MgCl_2_, 1% Triton X-100, 1 mM DTT, 0.1 mg/ml CHX, 1X proteinase inhibitor cocktail (Sigma-Aldrich), and 1 mM PMSF). The lysates were centrifuged at 12,000 x g for 10 min at 4°C and 1/10 of the supernatant was removed as input sample. The remainder of each supernatant was loaded on a 20%–50% sucrose gradient prepared in PLB (without Triton X-100) and resolved by centrifugation in an SW41 rotor (Beckman) at 38,000 rpm for 3 h at 4°C. Fractions were collected while monitoring the absorbance at 254 nm. RNA was prepared from each fraction and assayed by quantitative RT-PCR.

### Quantification and statistical analysis

Statistical details for each experiment, including *n* numbers and the statistical test performed, can be found in the corresponding figure legend. Data are presented as mean ± standard error of the mean (SEM). Statistical analysis was performed using Prism 6 software. Student’s t test was used for datasets with a normal distribution and a single intervention. One-way ANOVA was performed with Tukey’s post hoc test for multiple comparisons, and two-way ANOVA with Sidak’s post hoc test was used to compare ribosome loading of *erk* mRNA. *p* < 0.05 was considered statistically significant. *p < 0.05, **p < 0.01, ***p < 0.001, ****p < 0.0001.

## Supporting information

S1 Fig Genetic screen for suppressors of general polyQ model in fly eyes. (A) Light microscope images of eyes of 1-day-old wild-type and *GMR-63Q* (*ey-flp GMR-63Q-HA*) flies. Scale bar: 50 μm. (B) Transmission electron microscopy (TEM) images of 1-day-old flies from wild-type and *GMR-63Q* eyes. Scale bar: 2 μm. (C) Eye imaginal discs (left panels) and pupa eye (right panels) of *GMR-63Q* and *GMR-31Q* flies were stained against anti-HA antibody and anti-Myc antibody, respectively. A: anterior area, P: posterior area. Scale bar: 10 μm. (D) EMS screening strategy to identify suppressors of 63Q induced cell death. Take the screening of the second chromosome as an example. The second chromosome of *ey-flp;FRT40A* flies was isogenized. Flies by feeding 25 mM EMS (Sigma) in 2% sucrose for 8 h, followed by mating to *ey-flp GMR-63Q-HA;GMR-hid CL FRT40A/CyO hs-hid* flies. Flies were heat shocked at 37°C for 1 h to avoid having heterozygous flies among the F1 progeny (the pro-apoptosis gene *hid* was induced by heat-shock (*hs-hid*) in heterozygous flies). Suppression of retinal cell death in 1-day-old male flies were accessed. For other chromosomal arms, the isogenized *ey-flp;FRT42D*, *ey-flp;FRT2A and ey-flp;FRT82B* flies were mutagenized, followed by crossing with *ey-flp GMR-63Q-HA;FRT42D GMR-hid CL /CyO hs-hid*, *ey-flp GMR-63Q-HA;GMR-hid CL FRT2A/TM3 hs-hid* flies and *ey-flp GMR-63Q-HA;FRT82B GMR-hid CL/TM3 hs-hid* flies, respectively.
(TIF)

S2 Fig The *rack1* mutants specifically suppress polyQ induced cell death. (A) Western blot analysis of proteins extracted from wild type and *rack1*^*s12*^ (*rack1*^*s12*^ *FRT40A/GMR-hid CL FRT40A*) fly eyes with antibody against RACK1. β-Actin was used as loading control. (B) TEM images of 1-day-old flies from wild type, *MJD-Q78* (*GMR-GAL4/+;UAS-MJD*.*tr-Q78 /+*), and *MJD-Q78 rack1*^*RNAi*^ (*GMR-GAL4/+;UAS-MJD*.*tr-Q78 /UAS-rack1*^*GD12135*^) eyes. Scale bar: 2 μm. (C) TEM images of wild type, *GMR>Htt96Q* (*GMR-GAL4/+;UAS-Htt96Q-eGFP/+*) and *GMR>Htt96Q*, *rack1*^*RNAi*^ (*GMR-GAL4/+;UAS-Htt96Q-eGFP/UAS-rack1*^*GD12135*^) fly eyes at indicated days. Scale bar: 2 μm. (D) Light microscope images of eyes of 1-day-old wild-type (*GMR-GAL4/+*), *Rh1*^*G69D*^ (*GMR-GAL4 UAS-Rh1*^*G69D*^*/+*), *Rh1*^*G69D*^ *rack1*^*RNAi*^ (*GMR-GAL4 UAS-Rh1*^*G69D*^*/rack1*^*KK109073*^), *Tau*^*V337M*^ (*GMR-Tau*^*V337M*^*/+*), and *Tau*^*V337M*^, *rack1*^*RNAi*^ (*GMR-GAL4 UAS-rack1*^*KK109073*^*;GMR-Tau*^*V337M*^*/+*) flies. Scale bar: 50 μm. (E) Western blot analysis of Htt25Q proteins extracted from fly eyes with antibody against GFP. β-Actin was used as a loading control. (F) Fold-change of Htt25Q level in *GMR>Htt25Q*, *rack1*^*RNAi*^ (*GMR-GAL4/UAS-Htt25Q-eGFP;+/UAS-rack1*^*GD12135*^) flies compared to *GMR>Htt25Q* (*GMR-GAL4/UAS-Htt25Q-eGFP*) flies (n = 3). ns: not significant (Student’s unpaired *t* test). Data are presented as mean ±SEM.
(TIF)

S3 Fig Disruption of *rack1* suppresses polyQ induced cell death through a novel pathway. (A) RACK1 associated translational pathways are not involved in suppression of polyQ cytotoxicity. Light microscope images of eyes of 1-day-old flies expressing *GFP*, *pkc53e*^*RNAi*^, *pkc53e*, and *znf598*^*RNAi*^ or with deletion of *pkc53e* (*pkc53e*^*KO*^) in either *GMR-63Q;GMR-GAL4* (top panels), *GMR-63Q;GMR>rack1*^*RNAi*^ (middle panels), or *GMR-GAL4* (bottom panels) background. (B) RACK1 associated JNK/p38 pathways are not involved in suppression of polyQ induced cell death by *rack1* mutations. Light microscope images of eyes of 1-day-old flies expressing *p38b*^*RNAi*^, *p38b*, *jnk*^*RNAi*^, *JNK*, *p38a*^*RNAi*^, and *p38a* in either *GMR-63Q;GMR-GAL4* (top panels), *GMR-63Q;GMR>rack1*^*RNAi*^ (middle panels), or *GMR-GAL4* (bottom panels) background. (C) Apoptosis pathways are not involved in regulation of polyQ cytotoxicity by *rack1*. Light microscope images of eyes of 1-day-old flies of *Df(3L)H99 (*deletion of *rpr*, *grim*, and *hid)*, *UAS-rpr*^*RNAi*^, *UAS-diap1*, *UAS-dronc*^*RNAi*^, *UAS-dcp-1*^*RNAi*^, and *UAS-decay*^*RNAi*^ in combination with *GMR-63Q;GMR-GAL4* (top panels), *GMR-63Q;GMR>rack1*^*RNAi*^ (middle panels), or *GMR-GAL4* (bottom panels). Scale bar: 50 μm. Significant differences were determined by Two-way ANOVA with Sidak’s post hoc test (n = 3).
(TIF)

S4 Fig Aggregates of polyQ protein were unaffected by modification of *poe*, *kcmf1*, *usp47* and *erk*. (A) Pupa eyes of indicated genotypes were stained for 63Q-Myc using anti-Myc antibodies. The “flip-out” clones expressing *mCD8-GFP* (*hs-flp;GMR-63Q-Myc/+;UAS-mCD8-GFP/actin>>CD2>>GAL4 UAS-GFP/+*), *dnaJ-1* (*hs-flp;GMR-63Q-Myc/+;UAS-dnaJ-1/actin>>CD2>>GAL4 UAS-GFP*), *poe*^*RNAi*^ (*hs-flp;GMR-63Q-Myc/+;UAS-poe*^*HMS00739*^*/actin>>CD2>>GAL4 UAS-GFP*), *kcmf1*^*RNAi*^ (*hs-flp;GMR-63Q-Myc/+;UAS-kcmf1*^*HMS00511*^*/actin>>CD2>>GAL4 UAS-GFP*), *ufd4*^*RNAi*^ (*hs-flp;GMR-63Q-Myc/+;UAS-ufd4*^*RNAi*^*/actin>>CD2>>GAL4 UAS-GFP*), *USP47* (*hs-flp;GMR-63Q-Myc/+;UAS-USP47/actin>>CD2>>GAL4 UAS-GFP*), *erk*^*RNAi*^ (*hs-flp;GMR-63Q-Myc/+;UAS-erk*^*JF01366*^*/actin>>CD2>>GAL4 UAS-GFP*) *and erk* (*hs-flp;GMR-63Q-Myc/+;UAS-erk/actin>>CD2>>GAL4 UAS-GFP*) were generated by heat-shock. GFP positive clones are cells expressing indicated RNAi/genes. The *mCD8-GFP* and *dnaJ-1* expressing flies were served as negative and positive controls, respectively. Scale bar: 10 μm. (B) Quantification of 63Q relative fluorescence intensity (upper panel) and relative quantity of 63Q aggregates (lower panel) between mutant cells and control cells from indicated genotypes. Data are presented as mean ± standard error of the mean (SEM). Significant differences were determined by one-way ANOVA with Tukey’s post hoc test (n = 3). (C-D) Expression of ERK is unaffected by loss of *rack1*.
(TIF)

S5 Fig Knocking down *rack1*, *poe*, or *kcmf1* increased ERK levels in the 63Q background, whereas knocking down *ufd4* did not affect ERK levels. (A) Pupa eyes of indicated genotypes were stained for ERK and 63Q-Myc using anti-ERK and anti-Myc antibodies, respectively. The “flip-out” clones expressing *GFP* (*hs-flp;GMR-63Q-Myc/+;UAS-GFP/actin>>CD2>>GAL4 UAS-GFP/+*), *rack1*^*RNAi*^ (*hs-flp;GMR-63Q-Myc/+;UAS- rack1*^*GD12135*^*/actin>>CD2>>GAL4 UAS-GFP*), *poe*^*RNAi*^ (*hs-flp;GMR-63Q-Myc/+;UAS-poe*^*HMS00739*^*/actin>>CD2>>GAL4 UAS-GFP*), *kcmf1*^*RNAi*^ (*hs-flp;GMR-63Q-Myc/+;UAS-kcmf1*^*HMS00511*^*/actin>>CD2>>GAL4 UAS-GFP*), *and ufd4*^*RNAi*^ (*hs-flp;GMR-63Q-Myc/+;UAS-ufd4*^*RNAi*^*/actin>>CD2>>GAL4 UAS-GFP*) were generated by heat-shock. GFP positive clones are cells expressing indicated RNAis. The *GFP* expressing flies were served as negative control. Scale bar: 10 μm. (B) Quantification of ERK relative fluorescence intensity between mutant cells and control cells from indicated genotypes. Data are presented as mean ± standard error of the mean (SEM). Significant differences were determined by one-way ANOVA with Tukey’s post hoc test (n = 3).
(TIF)

S6 Fig RACK1 regulates ERK post-translationally. (A) The *erk* mRNA levels are measured by qPCR in indicated fly eyes (normalized to *GMR-Gal4* flies, n = 3). Data are presented as mean ± standard error of the mean (SEM). (B) Polysomal loading of *erk* transcripts is not altered by *rack1* depletion. S2 cells were treated with *GFP* or *rack1* dsRNAs, and the polysome and monosome fractions were separated on a sucrose gradient. qPCR was used to assay *erk* mRNA transcript levels in both fractions. Data are presented as mean ± standard error of the mean (SEM). (C) Western blot analysis of proteins extracted from pupa eyes of wild-type (*GMR-GAL4/+*), *GMR-63Q* (*GMR-63Q-HA*), *MJD-78Q* (*GMR-GAL4/+;UAS-MJD*.*tr-Q78/+*) flies with antibody against ERK. β-Actin was used as an internal control. (D) Quantification of ERK levels in (C). Data are represented as mean ± SEM, and one-way ANOVA with Tukey’s post hoc test is used (n = 4).
(TIF)

S7 Fig RACK1 regulates the interaction between ERK and POE. (A) Schematic representations of POE protein with the position of identifiable domains and regions. UBR: ubiquitin protein ligase E3 component n-recognin domain; DZR: double zinc ribbon domain; UBR4: E3 ubiquitin ligase domain. Different mCherry-tagged portions of POE are presented below with their respective amino acids (a.a.) positions. (B) RACK1 interacts with 1864–2374 and 3377–4492 mCherry-tagged POE. Truncate POE-mCherry was co-expressed in S2 cells with GFP or RACK1-GFP, followed by immunoprecipitation with anti-GFP antibody. (C) S2 cells were transfected with GFP or RACK1-GFP for two days, followed by co-expression of different mCherry-tagged portions of POE and FLAG-tagged ERK. Cells were lysed and immunoprecipitated with anti-mCherry antibody. (D) Pupa eyes of indicated genotypes were stained for ERK and 63Q-Myc using anti-ERK and anti-Myc antibodies, respectively. The “flip-out” clones expressing *GFP* (*hs-flp;GMR-63Q-Myc/+;UAS-GFP/actin>>CD2>>GAL4 UAS-GFP/+*), *rack1*^*RNAi*^ *kcmf1*^*OE*^ (*hs-flp;GMR-63Q-Myc/UAS-rack1*^*KK109073*^*;UAS-kcmf1/actin>>CD2>>GAL4 UAS-GFP*) and *rack1*^*RNAi*^ *poe*^*OE*^ (*hs-flp;GMR-63Q-Myc/UAS-rack1*^*KK109073*^*;UAS-poe/actin>>CD2>>GAL4 UAS-GFP*) were generated by heat-shock. GFP positive clones are cells expressing indicated RNAi/genes. The *GFP* expressing flies were served as negative control. Scale bar: 10 μm. (E) Quantification of ERK relative fluorescence intensity between mutant cells and control cells from indicated genotypes. Data are presented as mean ± standard error of the mean (SEM). Significant differences were determined by one-way ANOVA with Tukey’s post hoc test (n = 3).
(TIF)

S8 Fig Quantitative analysis of eye phenotypes. (A) quantification of eye degeneration of flies in Fig 1A. (B) quantification of eye degeneration of flies in Fig 3A. (C) quantification of eye degeneration of flies in Fig 6D. (D) quantification of eye degeneration of flies in Fig 7F. Data are presented as mean ± standard error of the mean (SEM). Significant differences were determined by one-way ANOVA with Tukey’s post hoc test (n = 50). Details of quantification criteria are demonstrated in Methods and Materials.
(TIF)

S1 Table qPCR validation of indicated RNAi lines. *UAS-RNAi* lines were crossed with *GMR-GAL4*, and adult eyes of F1 flies were dissected for RNA extraction and subsequent qPCR. Samples were prepared in triplicate. All values were normalized to *GMR-GAL4* samples. RpL32 was used as the reference gene for standardization. The p-values were obtained using a Student’s T-tests against the *GMR-GAL4* samples.
(XLSX)

S1 Data Original data statistics. (XLSX)
