# Supplementary figures and images for: RACK1 modulates polyglutamine-induced neurodegeneration by promoting ERK degradation in Drosophila
Source: PLoS Genet. 2021 May 13;17(5):e1009558. doi: 10.1371/journal.pgen.1009558 (PMC8118270; doi:10.1371/journal.pgen.1009558)

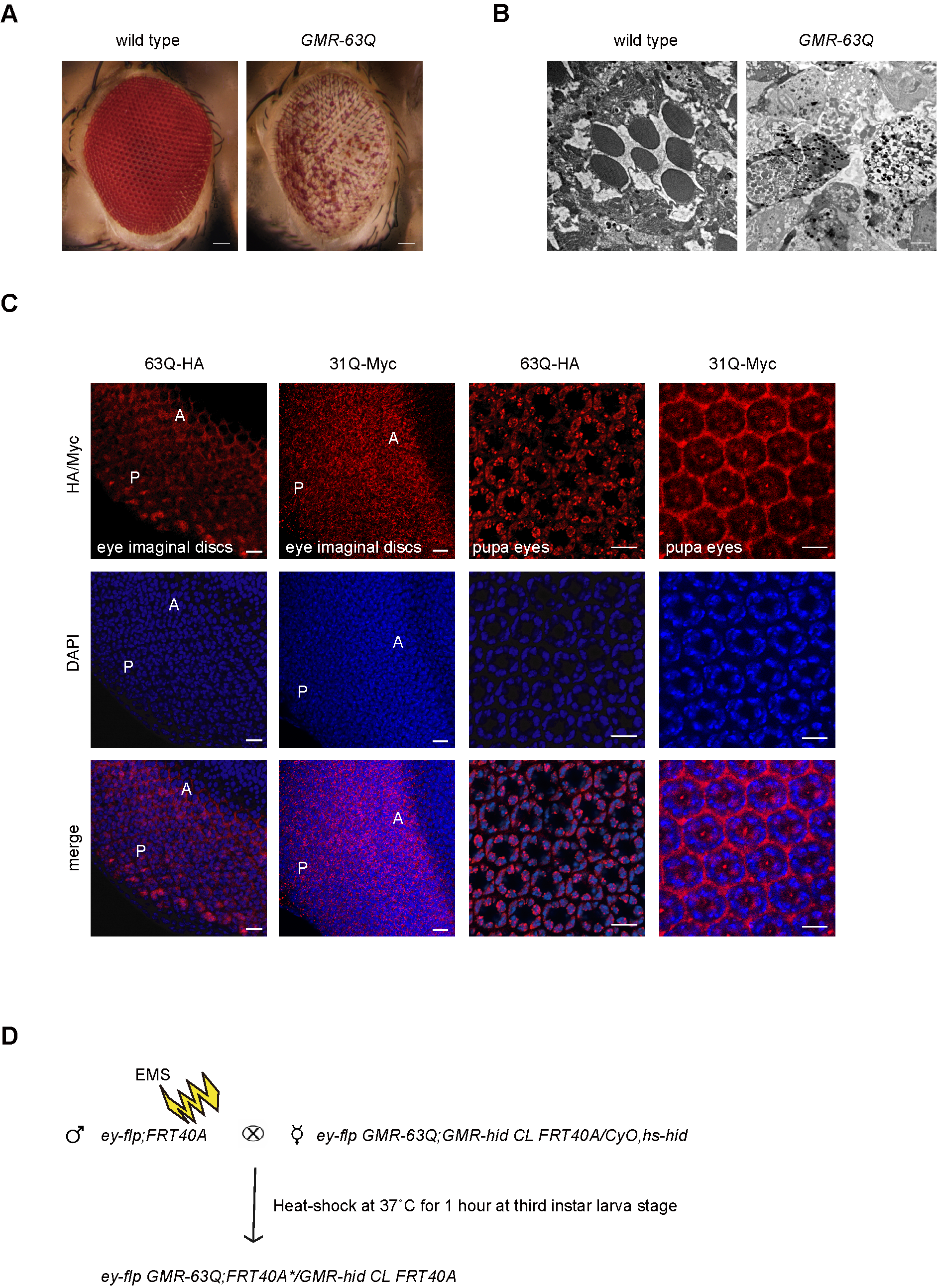

Supplement: S1 Fig — (A) Light microscope images of eyes of 1-day-old wild-type and GMR-63Q (ey-flp GMR-63Q-HA) flies. Scale bar: 50 μm. (B) Transmission electron microscopy (TEM) images of 1-day-old flies from wild-type and GMR-63Q eyes. Scale bar: 2 μm. (C) Eye imaginal discs (left panels) and pupa eye (right panels) of GMR-63Q and GMR-31Q flies were stained against anti-HA antibody and anti-Myc antibody, respectively. A: anterior area, P: posterior area. Scale bar: 10 μm. (D) EMS screening strategy to identify suppressors of 63Q induced cell death. Take the screening of the second chromosome as an example. The second chromosome of ey-flp;FRT40A flies was isogenized. Flies by feeding 25 mM EMS (Sigma) in 2% sucrose for 8 h, followed by mating to ey-flp GMR-63Q-HA;GMR-hid CL FRT40A/CyO hs-hid flies. Flies were heat shocked at 37°C for 1 h to avoid having heterozygous flies among the F1 progeny (the pro-apoptosis gene hid was induced by heat-shock (hs-hid) in heterozygous flies). Suppression of retinal cell death in 1-day-old male flies were accessed. For other chromosomal arms, the isogenized ey-flp;FRT42D, ey-flp;FRT2A and ey-flp;FRT82B flies were mutagenized, followed by crossing with ey-flp GMR-63Q-HA;FRT42D GMR-hid CL /CyO hs-hid, ey-flp GMR-63Q-HA;GMR-hid CL FRT2A/TM3 hs-hid flies and ey-flp GMR-63Q-HA;FRT82B GMR-hid CL/TM3 hs-hid flies, respectively. (TIF) [file pgen.1009558.s001.tif]

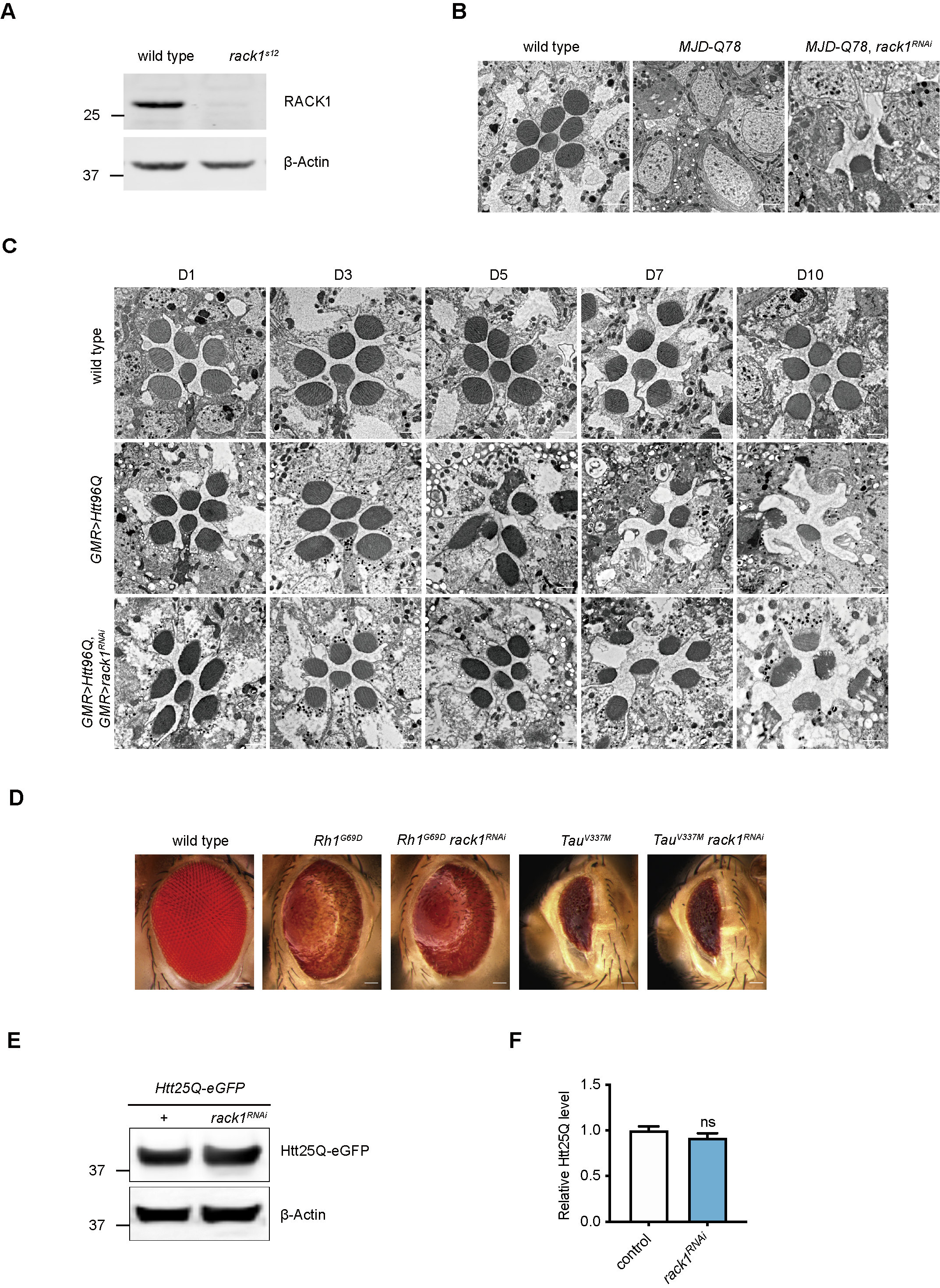

Supplement: S2 Fig — (A) Western blot analysis of proteins extracted from wild type and rack1s12 (rack1s12 FRT40A/GMR-hid CL FRT40A) fly eyes with antibody against RACK1. β-Actin was used as loading control. (B) TEM images of 1-day-old flies from wild type, MJD-Q78 (GMR-GAL4/+;UAS-MJD.tr-Q78 /+), and MJD-Q78 rack1RNAi (GMR-GAL4/+;UAS-MJD.tr-Q78 /UAS-rack1GD12135) eyes. Scale bar: 2 μm. (C) TEM images of wild type, GMR>Htt96Q (GMR-GAL4/+;UAS-Htt96Q-eGFP/+) and GMR>Htt96Q, rack1RNAi (GMR-GAL4/+;UAS-Htt96Q-eGFP/UAS-rack1GD12135) fly eyes at indicated days. Scale bar: 2 μm. (D) Light microscope images of eyes of 1-day-old wild-type (GMR-GAL4/+), Rh1G69D (GMR-GAL4 UAS-Rh1G69D/+), Rh1G69D rack1RNAi (GMR-GAL4 UAS-Rh1G69D/rack1KK109073), TauV337M (GMR-TauV337M/+), and TauV337M, rack1RNAi (GMR-GAL4 UAS-rack1KK109073;GMR-TauV337M/+) flies. Scale bar: 50 μm. (E) Western blot analysis of Htt25Q proteins extracted from fly eyes with antibody against GFP. β-Actin was used as a loading control. (F) Fold-change of Htt25Q level in GMR>Htt25Q, rack1RNAi (GMR-GAL4/UAS-Htt25Q-eGFP;+/UAS-rack1GD12135) flies compared to GMR>Htt25Q (GMR-GAL4/UAS-Htt25Q-eGFP) flies (n = 3). ns: not significant (Student’s unpaired t test). Data are presented as mean ±SEM. (TIF) [file pgen.1009558.s002.tif]

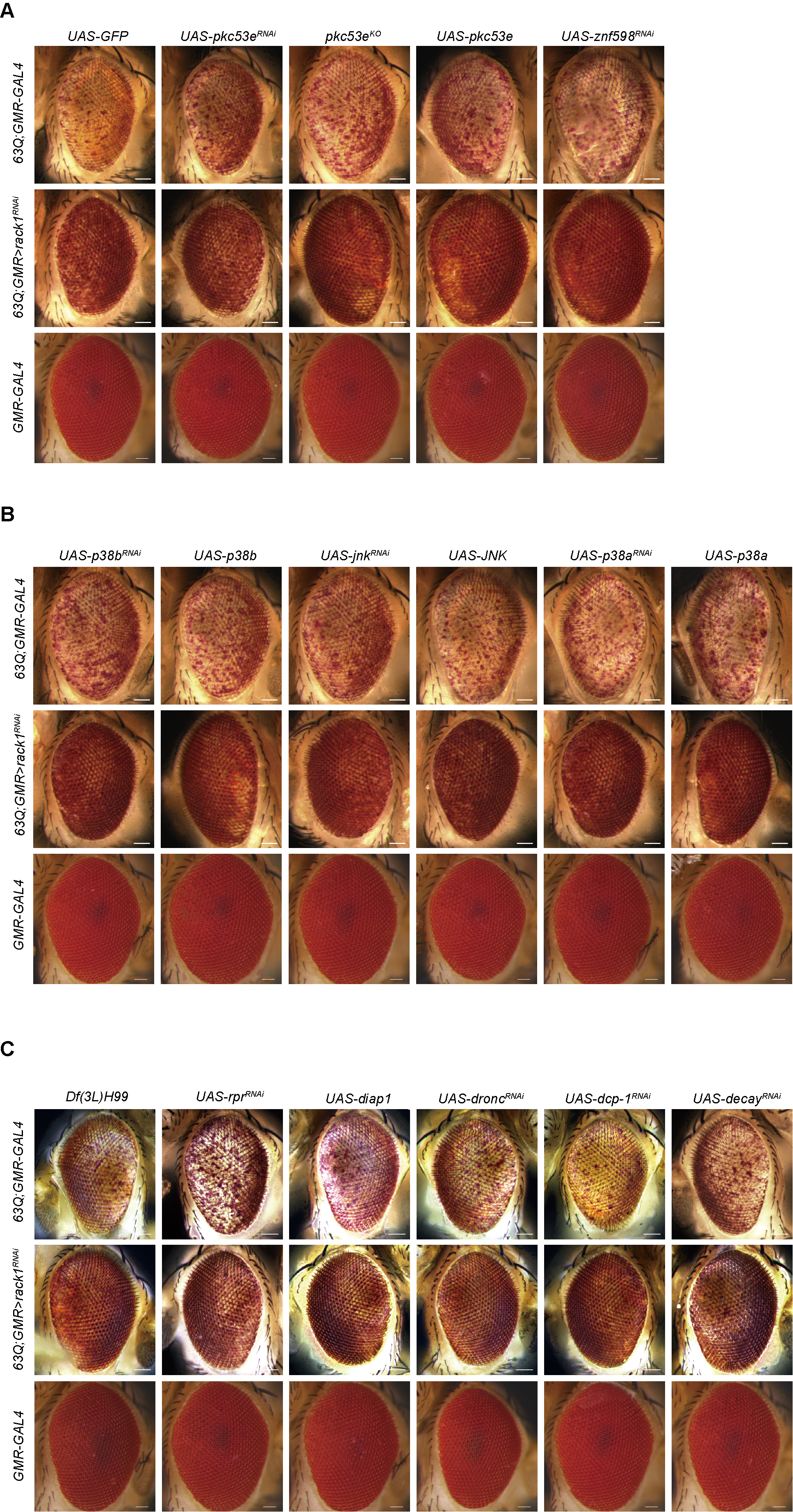

Supplement: S3 Fig — (A) RACK1 associated translational pathways are not involved in suppression of polyQ cytotoxicity. Light microscope images of eyes of 1-day-old flies expressing GFP, pkc53eRNAi, pkc53e, and znf598RNAi or with deletion of pkc53e (pkc53eKO) in either GMR-63Q;GMR-GAL4 (top panels), GMR-63Q;GMR>rack1RNAi (middle panels), or GMR-GAL4 (bottom panels) background. (B) RACK1 associated JNK/p38 pathways are not involved in suppression of polyQ induced cell death by rack1 mutations. Light microscope images of eyes of 1-day-old flies expressing p38bRNAi, p38b, jnkRNAi, JNK, p38aRNAi, and p38a in either GMR-63Q;GMR-GAL4 (top panels), GMR-63Q;GMR>rack1RNAi (middle panels), or GMR-GAL4 (bottom panels) background. (C) Apoptosis pathways are not involved in regulation of polyQ cytotoxicity by rack1. Light microscope images of eyes of 1-day-old flies of Df(3L)H99 (deletion of rpr, grim, and hid), UAS-rprRNAi, UAS-diap1, UAS-droncRNAi, UAS-dcp-1RNAi, and UAS-decayRNAi in combination with GMR-63Q;GMR-GAL4 (top panels), GMR-63Q;GMR>rack1RNAi (middle panels), or GMR-GAL4 (bottom panels). Scale bar: 50 μm. Significant differences were determined by Two-way ANOVA with Sidak’s post hoc test (n = 3). (TIF) [file pgen.1009558.s003.tif]

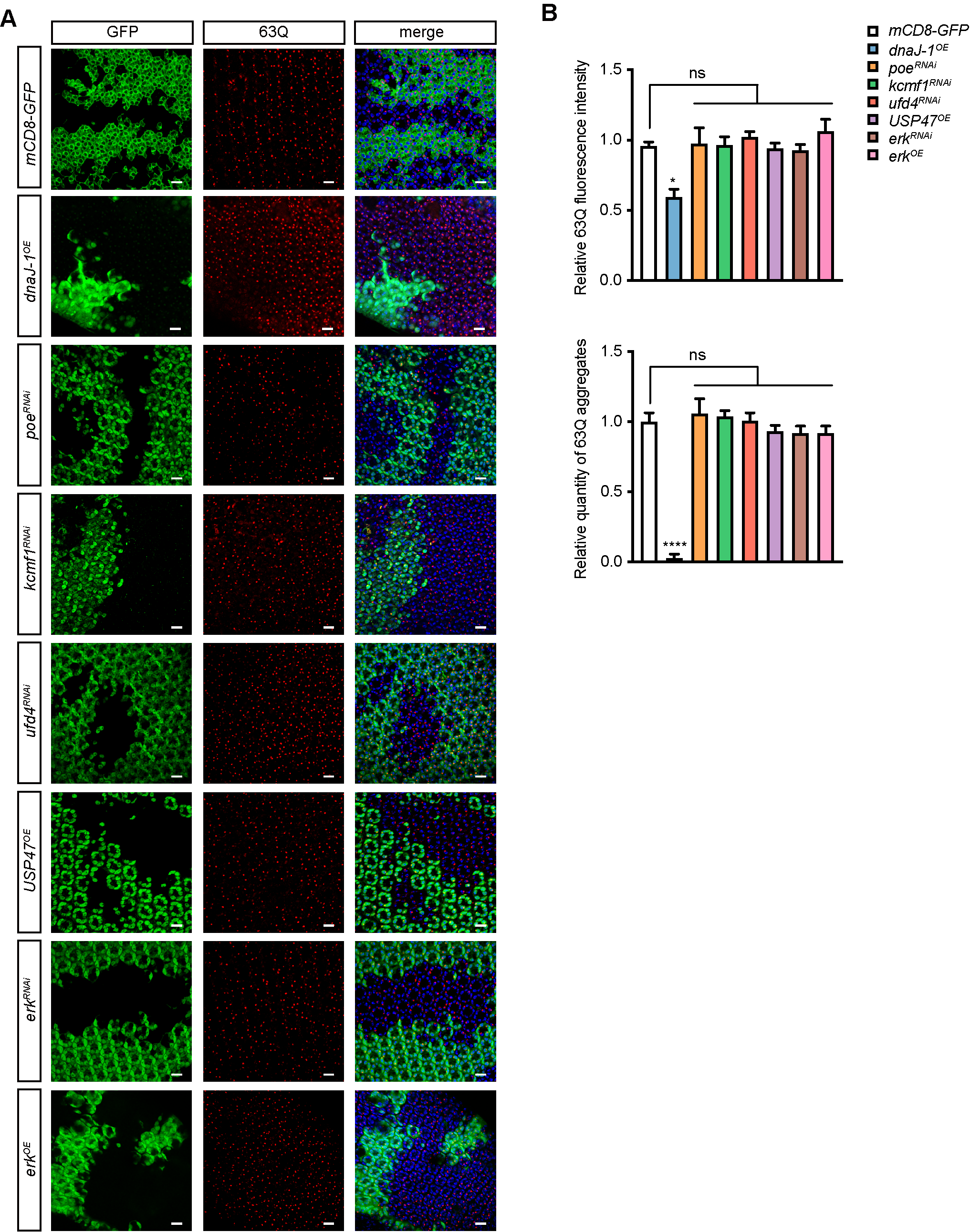

Supplement: S4 Fig — (A) Pupa eyes of indicated genotypes were stained for 63Q-Myc using anti-Myc antibodies. The “flip-out” clones expressing mCD8-GFP (hs-flp;GMR-63Q-Myc/+;UAS-mCD8-GFP/actin>>CD2>>GAL4 UAS-GFP/+), dnaJ-1 (hs-flp;GMR-63Q-Myc/+;UAS-dnaJ-1/actin>>CD2>>GAL4 UAS-GFP), poeRNAi (hs-flp;GMR-63Q-Myc/+;UAS-poeHMS00739/actin>>CD2>>GAL4 UAS-GFP), kcmf1RNAi (hs-flp;GMR-63Q-Myc/+;UAS-kcmf1HMS00511/actin>>CD2>>GAL4 UAS-GFP), ufd4RNAi (hs-flp;GMR-63Q-Myc/+;UAS-ufd4RNAi/actin>>CD2>>GAL4 UAS-GFP), USP47 (hs-flp;GMR-63Q-Myc/+;UAS-USP47/actin>>CD2>>GAL4 UAS-GFP), erkRNAi (hs-flp;GMR-63Q-Myc/+;UAS-erkJF01366/actin>>CD2>>GAL4 UAS-GFP) and erk (hs-flp;GMR-63Q-Myc/+;UAS-erk/actin>>CD2>>GAL4 UAS-GFP) were generated by heat-shock. GFP positive clones are cells expressing indicated RNAi/genes. The mCD8-GFP and dnaJ-1 expressing flies were served as negative and positive controls, respectively. Scale bar: 10 μm. (B) Quantification of 63Q relative fluorescence intensity (upper panel) and relative quantity of 63Q aggregates (lower panel) between mutant cells and control cells from indicated genotypes. Data are presented as mean ± standard error of the mean (SEM). Significant differences were determined by one-way ANOVA with Tukey’s post hoc test (n = 3). (C-D) Expression of ERK is unaffected by loss of rack1. (TIF) [file pgen.1009558.s004.tif]

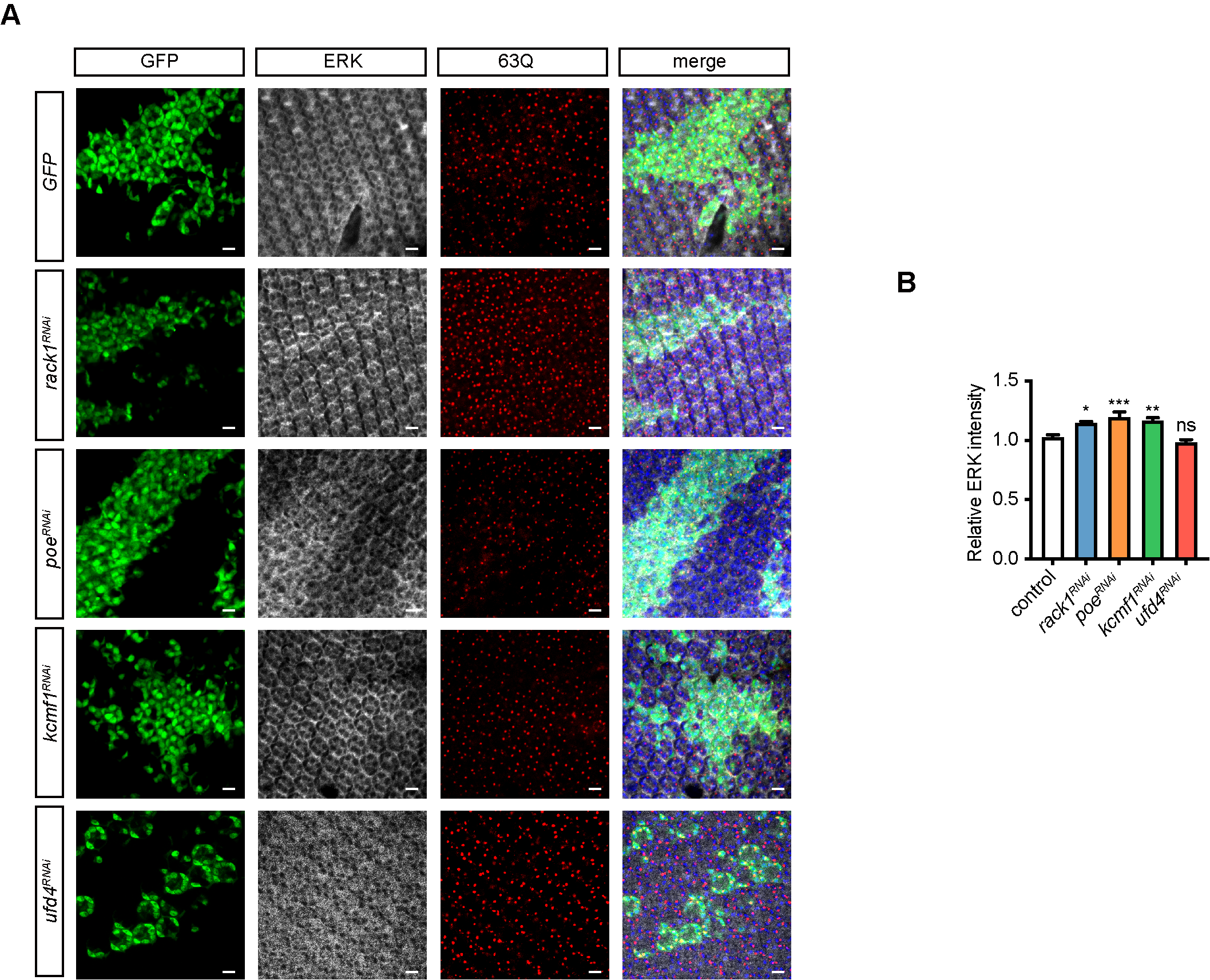

Supplement: S5 Fig — (A) Pupa eyes of indicated genotypes were stained for ERK and 63Q-Myc using anti-ERK and anti-Myc antibodies, respectively. The “flip-out” clones expressing GFP (hs-flp;GMR-63Q-Myc/+;UAS-GFP/actin>>CD2>>GAL4 UAS-GFP/+), rack1RNAi (hs-flp;GMR-63Q-Myc/+;UAS- rack1GD12135/actin>>CD2>>GAL4 UAS-GFP), poeRNAi (hs-flp;GMR-63Q-Myc/+;UAS-poeHMS00739/actin>>CD2>>GAL4 UAS-GFP), kcmf1RNAi (hs-flp;GMR-63Q-Myc/+;UAS-kcmf1HMS00511/actin>>CD2>>GAL4 UAS-GFP), and ufd4RNAi (hs-flp;GMR-63Q-Myc/+;UAS-ufd4RNAi/actin>>CD2>>GAL4 UAS-GFP) were generated by heat-shock. GFP positive clones are cells expressing indicated RNAis. The GFP expressing flies were served as negative control. Scale bar: 10 μm. (B) Quantification of ERK relative fluorescence intensity between mutant cells and control cells from indicated genotypes. Data are presented as mean ± standard error of the mean (SEM). Significant differences were determined by one-way ANOVA with Tukey’s post hoc test (n = 3). (TIF) [file pgen.1009558.s005.tif]

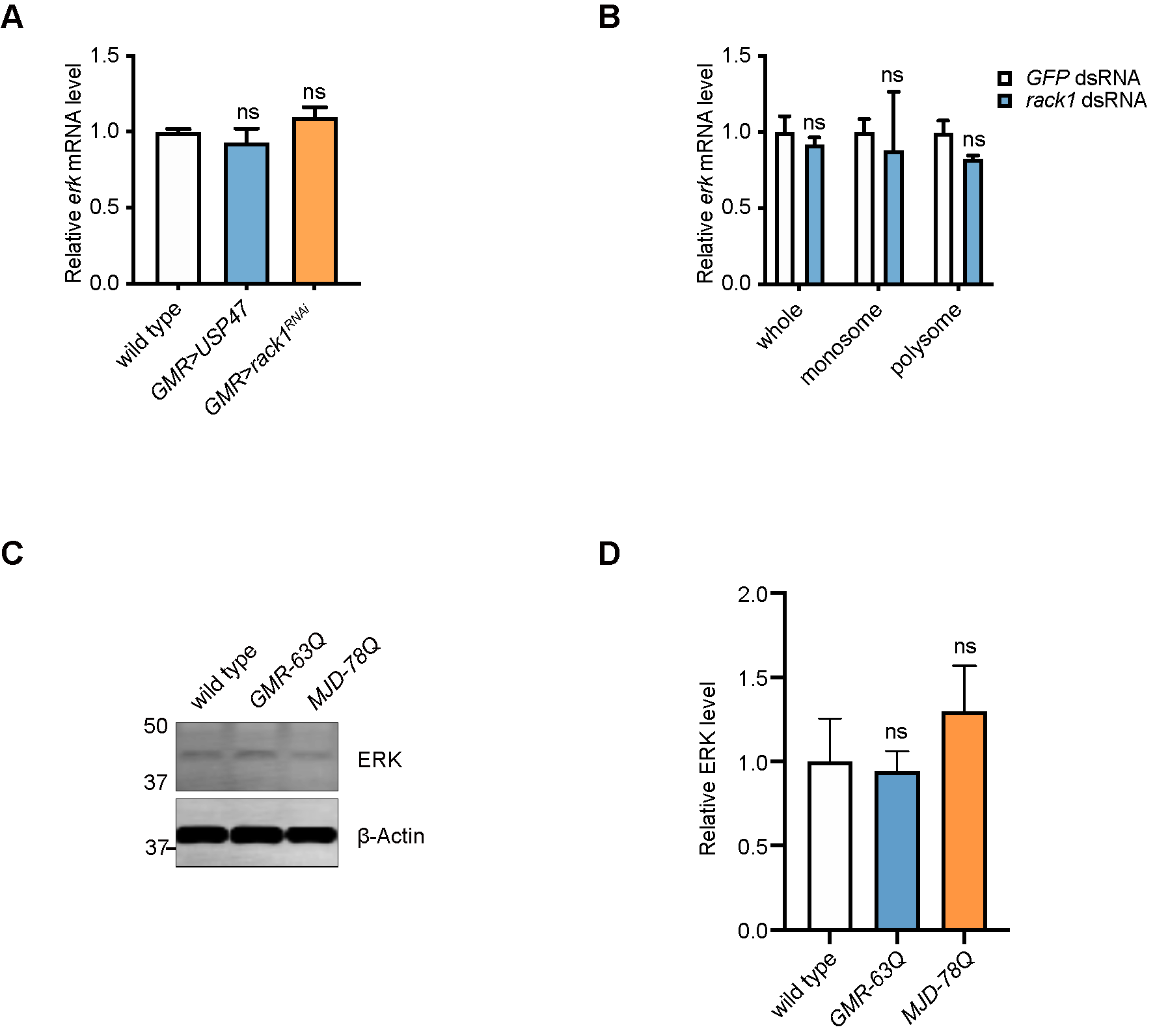

Supplement: S6 Fig — (A) The erk mRNA levels are measured by qPCR in indicated fly eyes (normalized to GMR-Gal4 flies, n = 3). Data are presented as mean ± standard error of the mean (SEM). (B) Polysomal loading of erk transcripts is not altered by rack1 depletion. S2 cells were treated with GFP or rack1 dsRNAs, and the polysome and monosome fractions were separated on a sucrose gradient. qPCR was used to assay erk mRNA transcript levels in both fractions. Data are presented as mean ± standard error of the mean (SEM). (C) Western blot analysis of proteins extracted from pupa eyes of wild-type (GMR-GAL4/+), GMR-63Q (GMR-63Q-HA), MJD-78Q (GMR-GAL4/+;UAS-MJD.tr-Q78/+) flies with antibody against ERK. β-Actin was used as an internal control. (D) Quantification of ERK levels in (C). Data are represented as mean ± SEM, and one-way ANOVA with Tukey’s post hoc test is used (n = 4). (TIF) [file pgen.1009558.s006.tif]

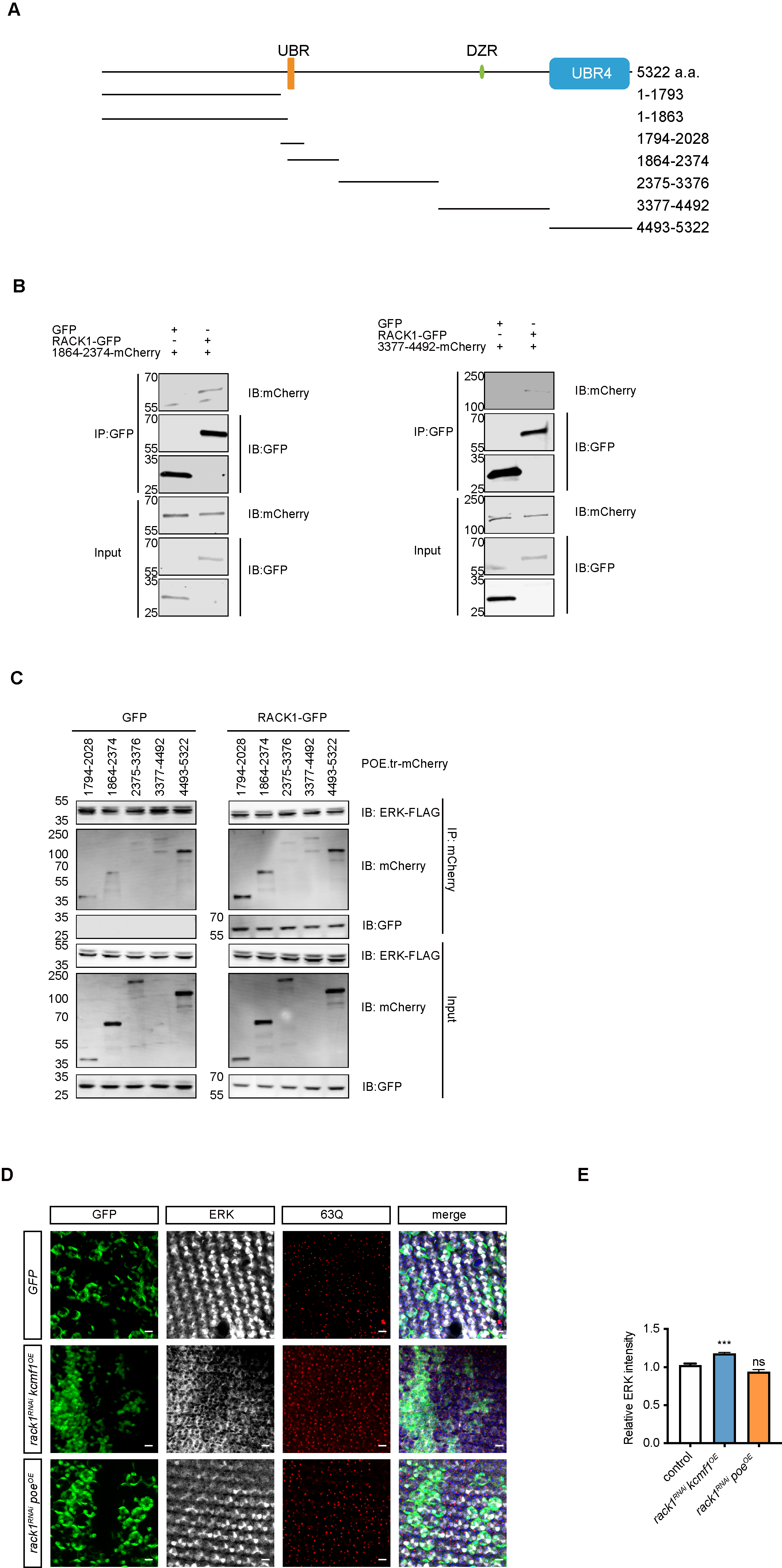

Supplement: S7 Fig — (A) Schematic representations of POE protein with the position of identifiable domains and regions. UBR: ubiquitin protein ligase E3 component n-recognin domain; DZR: double zinc ribbon domain; UBR4: E3 ubiquitin ligase domain. Different mCherry-tagged portions of POE are presented below with their respective amino acids (a.a.) positions. (B) RACK1 interacts with 1864–2374 and 3377–4492 mCherry-tagged POE. Truncate POE-mCherry was co-expressed in S2 cells with GFP or RACK1-GFP, followed by immunoprecipitation with anti-GFP antibody. (C) S2 cells were transfected with GFP or RACK1-GFP for two days, followed by co-expression of different mCherry-tagged portions of POE and FLAG-tagged ERK. Cells were lysed and immunoprecipitated with anti-mCherry antibody. (D) Pupa eyes of indicated genotypes were stained for ERK and 63Q-Myc using anti-ERK and anti-Myc antibodies, respectively. The “flip-out” clones expressing GFP (hs-flp;GMR-63Q-Myc/+;UAS-GFP/actin>>CD2>>GAL4 UAS-GFP/+), rack1RNAi kcmf1OE (hs-flp;GMR-63Q-Myc/UAS-rack1KK109073;UAS-kcmf1/actin>>CD2>>GAL4 UAS-GFP) and rack1RNAi poeOE (hs-flp;GMR-63Q-Myc/UAS-rack1KK109073;UAS-poe/actin>>CD2>>GAL4 UAS-GFP) were generated by heat-shock. GFP positive clones are cells expressing indicated RNAi/genes. The GFP expressing flies were served as negative control. Scale bar: 10 μm. (E) Quantification of ERK relative fluorescence intensity between mutant cells and control cells from indicated genotypes. Data are presented as mean ± standard error of the mean (SEM). Significant differences were determined by one-way ANOVA with Tukey’s post hoc test (n = 3). (TIF) [file pgen.1009558.s007.tif]

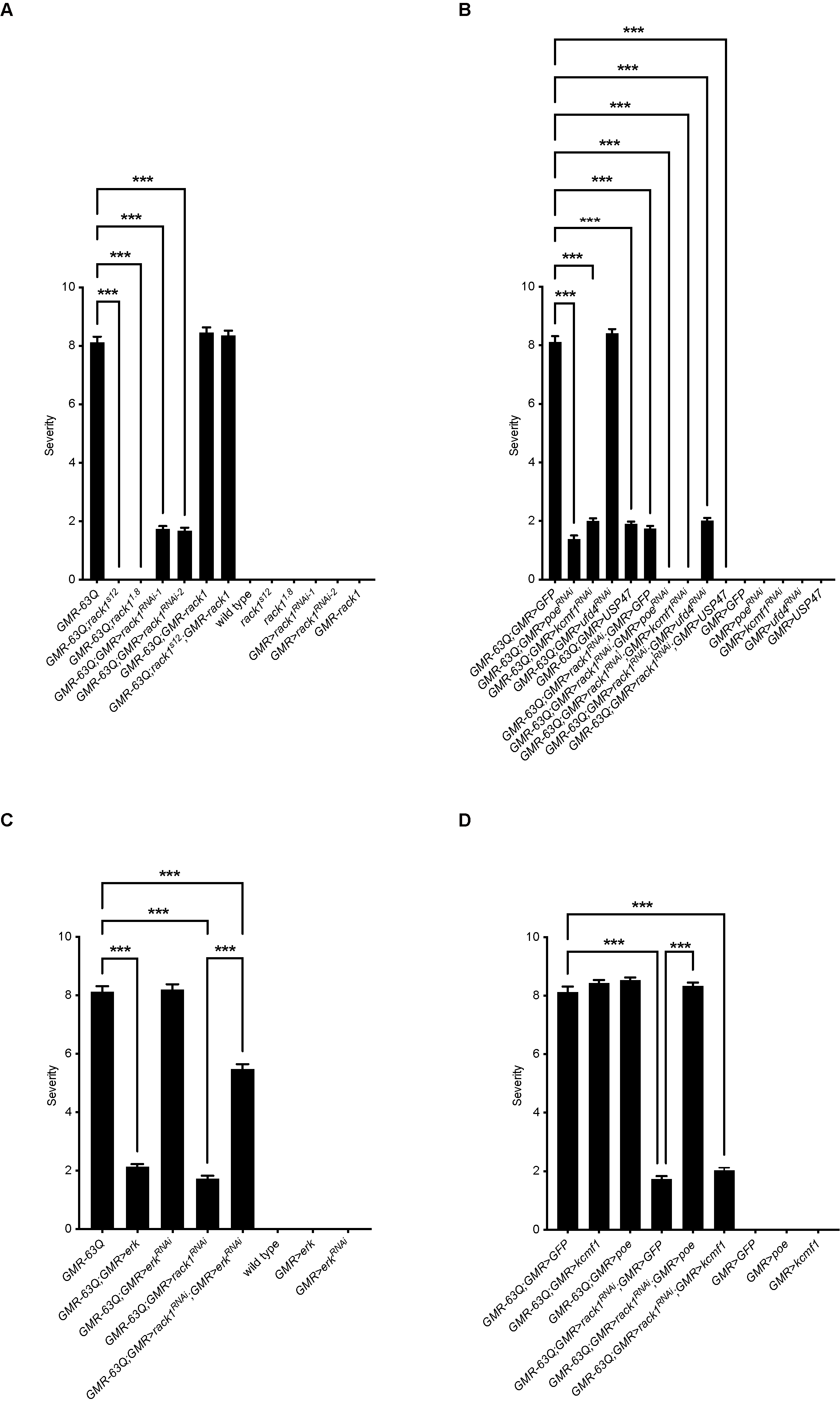

Supplement: S8 Fig — (A) quantification of eye degeneration of flies in Fig 1A. (B) quantification of eye degeneration of flies in Fig 3A. (C) quantification of eye degeneration of flies in Fig 6D. (D) quantification of eye degeneration of flies in Fig 7F. Data are presented as mean ± standard error of the mean (SEM). Significant differences were determined by one-way ANOVA with Tukey’s post hoc test (n = 50). Details of quantification criteria are demonstrated in Methods and Materials. (TIF) [file pgen.1009558.s008.tif]
